# Profiling of patients with type 2 diabetes based on medication adherence data

**DOI:** 10.3389/fpubh.2023.1209809

**Published:** 2023-07-06

**Authors:** Rene Markovič, Vladimir Grubelnik, Tadej Završnik, Helena Blažun Vošner, Peter Kokol, Matjaž Perc, Marko Marhl, Matej Završnik, Jernej Završnik

**Affiliations:** ^1^Faculty of Natural Sciences and Mathematics, University of Maribor, Maribor, Slovenia; ^2^Faculty of Electrical Engineering and Computer Science, University of Maribor, Maribor, Slovenia; ^3^University Clinical Medical Centre Maribor, Maribor, Slovenia; ^4^Faculty of Medicine, University of Maribor, Maribor, Slovenia; ^5^Community Healthcare Center Dr. Adolf Drolc Maribor, Maribor, Slovenia; ^6^Faculty of Health and Social Sciences, Slovenj Gradec, Slovenia; ^7^Alma Mater Europaea - ECM, Maribor, Slovenia; ^8^Complexity Science Hub Vienna, Vienna, Austria; ^9^Department of Medical Research, China Medical University Hospital, China Medical University, Taichung, Taiwan; ^10^Department of Physics, Kyung Hee University, Seoul, Republic of Korea; ^11^Faculty of Education, University of Maribor, Maribor, Slovenia; ^12^Department of Endocrinology and Diabetology, University Medical Center Maribor, Maribor, Slovenia; ^13^Science and Research Center Koper, Koper, Slovenia

**Keywords:** medication management, type 2 diabetes mellitus, patient profiles, cluster analysis, electronic health records, medication usage patterns, personalized medicine, natural language processing

## Abstract

**Introduction:**

Type 2 diabetes mellitus (T2DM) is a complex, chronic disease affecting multiple organs with varying symptoms and comorbidities. Profiling patients helps identify those with unfavorable disease progression, allowing for tailored therapy and addressing special needs. This study aims to uncover different T2DM profiles based on medication intake records and laboratory measurements, with a focus on how individuals with diabetes move through disease phases.

**Methods:**

We use medical records from databases of the last 20 years from the Department of Endocrinology and Diabetology of the University Medical Center in Maribor. Using the standard ATC medication classification system, we created a patient-specific drug profile, created using advanced natural language processing methods combined with data mining and hierarchical clustering.

**Results:**

Our results show a well-structured profile distribution characterizing different age groups of individuals with diabetes. Interestingly, only two main profiles characterize the early 40–50 age group, and the same is true for the last 80+ age group. One of these profiles includes individuals with diabetes with very low use of various medications, while the other profile includes individuals with diabetes with much higher use. The number in both groups is reciprocal. Conversely, the middle-aged groups are characterized by several distinct profiles with a wide range of medications that are associated with the distinct concomitant complications of T2DM. It is intuitive that the number of profiles increases in the later age groups, but it is not obvious why it is reduced later in the 80+ age group. In this context, further studies are needed to evaluate the contributions of a range of factors, such as drug development, drug adoption, and the impact of mortality associated with all T2DM-related diseases, which characterize these middle-aged groups, particularly those aged 55–75.

**Conclusion:**

Our approach aligns with existing studies and can be widely implemented without complex or expensive analyses. Treatment and drug use data are readily available in healthcare facilities worldwide, allowing for profiling insights into individuals with diabetes. Integrating data from other departments, such as cardiology and renal disease, may provide a more sophisticated understanding of T2DM patient profiles.

## 1. Introduction

Type 2 diabetes mellitus (T2DM) is a chronic disease affecting millions of people worldwide and its prevalence is increasing at an alarming rate (1). Scientific research into the profiling of individuals with diabetes aims to identify the factors that contribute to the development and progression of the disease and to develop personalized treatment strategies for patients. Several studies have examined the genetic, environmental, and lifestyle factors that play a role in the development of T2DM. These factors include obesity, physical inactivity, poor diet, genetic factors, and other associated conditions. By analyzing these factors in different patient populations, researchers have been able to identify subgroups of patients with similar characteristics who have a similar disease course and may benefit from specific treatment interventions. In health care in general, patient profiling has been used for a variety of purposes, including for grouping patients by risk of various diseases (2–4), prognosis and disease progression, tailoring medical therapy (5–7), identifying individuals who use health care services (8), and for identifying patient needs (9).

The performance of such profiling techniques largely relies on the availability of high-quality patient-specific records stored as electronic medical records (EMR) or electronic health records (EHR). In general, EMR are digital patient records and charts, while EHR include additional features such as tools for prescribing medications electronically, ordering labs, streamlining internal and external communications, and sharing data. Modern medical software generally includes an EHR, and the term is often used interchangeably with EMR (10). These records provide a comprehensive view of a patient’s health history, including diagnoses, medications, treatment plans, immunization dates, allergies, radiology images, and laboratory and test results (11). The use of EHR has been shown to improve patient care by providing accurate and legible notes that can track the individual’s lifetime of health status, medicine use, laboratory results, images from many sources, and transferable across locations (12). They also provide clinical guidelines, flag abnormal results, remind tests to be done, and reduce medication errors (12). Furthermore, the integration of artificial intelligence and machine learning techniques with EHR has the potential to enhance patient profiling.

Patient profiling allows us to more quickly find those for whom the disease course is unfavorable, and we can focus time, adjust therapy, and address special needs (13). A recent study, has for example shown that only a small percentage of patients with prediabetes remain in this condition, while the majority go on to develop diabetes or regress to normoglycemia, with distinct predictors for both directions (14). Patient profiling has been mainly performed using artificial intelligence, machine learning, and data mining, especially in cancer research (15, 16) and in personalized, predictive, and precision medicine (17–19). An interesting intelligent artificial tool that analyses patient health portfolios and supports patient profiling was presented by Gehani and Panda (20). Machine learning has been used to plan treatment pathways based on patient profiles (21). In addition to using traditional statistical clustering methods to assess patient profiles in diabetes, preferred communication channels have also been used and it has been shown, that effective interaction between healthcare providers and patients can lead to better treatment outcomes (22). In diabetes specifically, machine learning and data mining have been used to profile patients to predict diabetes-related complications (23) and to determine which combination therapy would be most appropriate for patients with a given profile to improve glycaemic outcomes (24).

Identifying subgroups and risk factors in individuals with diabetes can aid physicians in developing personalized treatment plans and interventions to reduce the risk of complications and improve patient outcomes (25, 26). Biomarkers such as HbA1c and hs-CRP have been shown to predict cardiovascular events in individuals with diabetes (27, 28). Albuminuria is also a predictor of cardiovascular events in individuals with diabetes (29, 30). Natriuretic peptides such as BNP and NT-proBNP are biomarkers for heart failure and have been shown to predict cardiovascular events in individuals with diabetes (31). Other potential biomarkers for T2DM include IL-37, IL-17A, and circular RNA (32, 33).

In the field of diabetology, there have been numerous attempts at profiling. For example, in the study by Aschner et al. (34), patient profiles related to diabetes care were related to track trends in glycemic control. Using patient profiles, it was shown that improved self-assessment in diabetes care can lead to improved glycemic control and sustained diabetes management (35). Profiles of individuals with diabetes have also been established to determine the best insulin combination for glycemic control (36). Profiling of individuals with diabetes has also been used to assess the risk of developing a diabetic foot ulcers (37). Sheehan et al. (37) looked for factors in profiling individuals with diabetes that would allow more efficient and faster access to a physician compared with a simple queue. Li et al. (38) used profiling to assess the risk of hospitalization and in-hospital mortality in individuals with diabetes. In addition, Zghebi et al. (39) developed a score based on 34 data routinely collected in the electronic health record to assess the risk of hospitalization and mortality in individuals with diabetes. Individuals with diabetes have also been profiled to achieve glycemic goals (40). Indeed, there is interindividual variability in drug response due to the presence of genetic polymorphisms that affect drug metabolism (41).

In diabetology, although various attempts have been made to profile individuals with diabetes, the efficacy of antidiabetic therapy is also influenced by interindividual variability in drug response (42–44). Although this field is still in its infancy, it is expected to bring further advances in relation to the current recommendations for phenotypic modulation of antidiabetic therapy (45–47). The aim of this study is to gain insight into the diverse profiles of individuals with diabetes based solely on the raw text documents used as Electronic Medical Records (EMR) created by their monitoring physicians. The primary purpose of these documents is to store patients’ historical data that is of interest to the physicians. The documents are not linked to any database or registry. We have developed an algorithm capable of extracting data on prescribed medications and laboratory data from blood and urine measurements from these documents through text analysis, which served as the main guide for the study. After training our language model, no other data sources were used in this study, only the documents created by the physicians, to create a chronologically structured dataset and perform patient profiling.

The manuscript is organized into five main sections, with additional information included in the Supplementary file. The Materials and Methods section provides a brief description of the methodology used for the study. A more detailed description of the methodology including the study design, data sources, text-mining techniques, patient profiling, and cluster extraction methods, and comparison of clusters between age groups, is provided in the Supplementary file. The Results section presents the research findings, with Section 3.1 focusing on the frequency and type of medications prescribed for different age groups, and Section 3.2 detailing the diabetic treatment profiles. This is followed by the Discussion section, in which we provide an analysis and interpretation of the study results and place the findings in the context of existing research. The last section, Conclusion, briefly summarizes the major findings of the study, and offers implications and recommendations based on the study results. Finally, the Supplementary file provides additional information about the study and details about the unsupervised patient group detection method used in the study and the hematologic characteristics of individuals with diabetes in different age groups.

## 2. Materials and methods

### 2.1. Study design and data sources

**Figure 1:**
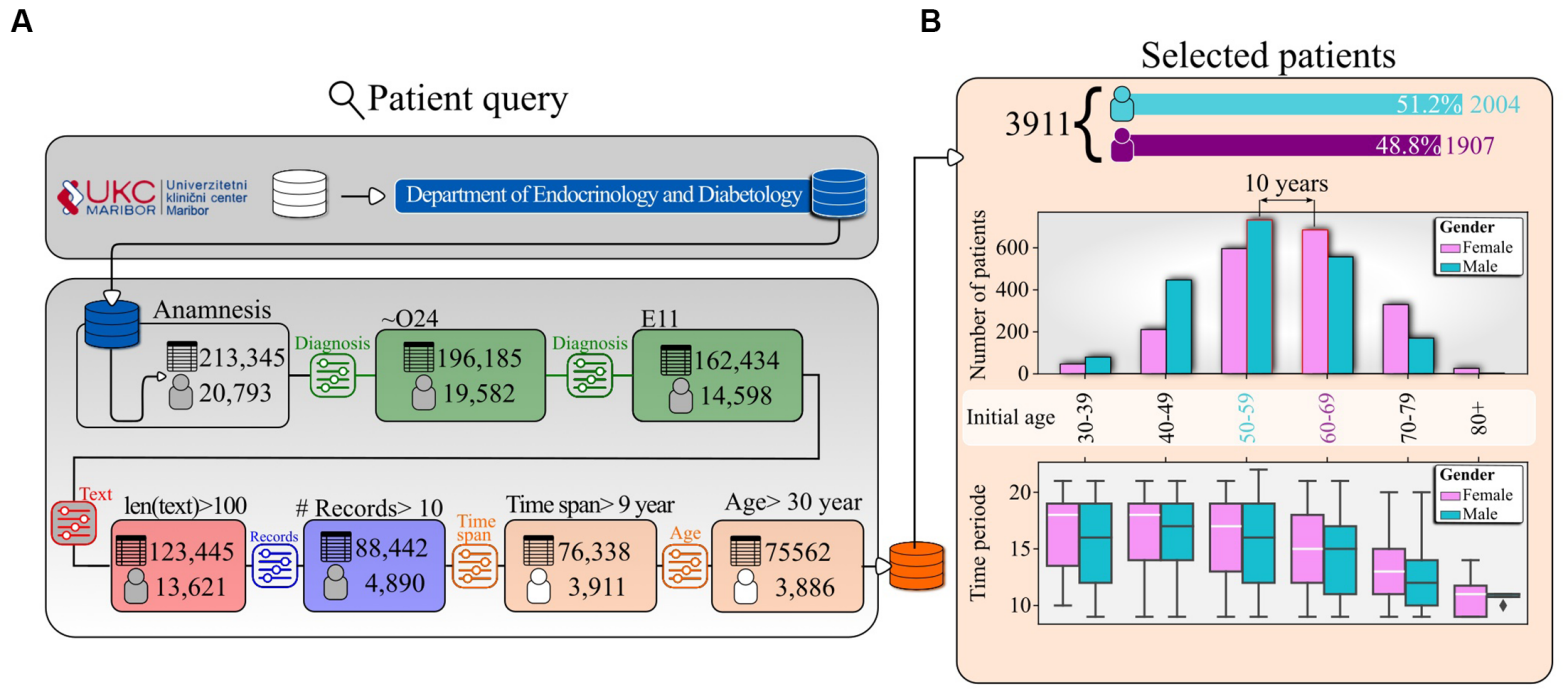
Data selection. **(A)** Patient query pipeline and **(B)** the number of male and female patients and the time span of the corresponding records by 10-year age groups. Patients were assigned to a specific age group based on age at initial presentation.

We obtained the data from the Maribor University Hospital (MUH) data center. The MUH staff provided us with a database of anonymized records of individuals with diabetes treated in the Department of Endocrinology and Diabetology (DED). The patient records were from 1997 to 2020, and the original dataset contained a total of 213,345 records for 20,793 different individuals with diabetes. Although the dataset obtained from the Data Centre was specifically queried for records created in DED, we add new criteria for extracting only individuals with diabetes. To do this, we examined up to 10 diagnoses if reported for a record in the dataset. Diagnoses were based on the International Classification of Diseases (ICD-11), although ICD-10 was also used for older records (48). First, we rejected all patients screened under the diagnosis O24 (diabetes mellitus (diabetes) during pregnancy). Next, we selected only records in which the diagnosis E11 (or a diagnosis derived from this branch of diagnosis) was reported as one of up to 10 diagnoses. Diagnosis E11 is T2DM. In addition, because we relied on the medical history text in our analyses, we selected only records in which the medical history text was longer than 100 characters. Finally, we excluded records belonging to patients who had been examined less than 10 times within a 10-year period and patients younger than 30 years. The result is the herein used database consisting of 75,562 records from 3,886 individuals with diabetes. To clarify the demographics of the final patient population, we also report basic demographic information on the number of patients who had their first screening at DED at a given age. Patients were divided into 10-year age groups (i.e., the 30–39 age group includes patients aged 30–39 years, inclusive). In addition, we also report the distribution of time periods during which patients in the corresponding age groups were continuously screened. The median follow-up duration of the individuals with diabetes is 15.4 years, with a range from the 5th percentile at 9.6 years to the 95th percentile at 19.8 years (see Supplementary file). The process of patient selection and the corresponding demographic characteristics are shown in Figure 1.

It should be noted that the texts from the documents used in this study are EMR created by physicians to track the progress of their patients. Hence, we needed to develop a text manning algorithm to extract relevant information from the EMR and to structure the extracted information’s in a form suitable for further processing and analysis. To extract that medication information from the patients’ EMR, we developed a novel natural language processing (NLP) algorithm and used knowledge from other sources. For example, we used the Central Drug Database (CDD) to identify medications and their corresponding Anatomical Therapeutic Chemical (ATC) codes. We focused on the ATC codes rather than the names of the drugs due to the hierarchical nature of the ATC classification system, which groups drugs based on their active ingredient and therapeutic purpose.

For each patient, we created a profile that reflected the range of medications they took during their diabetes treatment. We then computed a distance matrix between any pair of patients within a given age group based on their medication profiles. This matrix was used to capture the similarity of profiles and served as the basis for extracting homogeneous groups of individuals with diabetes treated in a similar way in an unsupervised manner using hierarchical clustering.

Once clusters within an age group were calculated, we compared them with clusters in adjacent age groups based on the similarity of their medication use profiles. This allowed us to establish a temporal link between two clusters in two adjacent age groups and track changes in medication use patterns over time.

This methodology allowed us to create patient-specific profiles, identify clusters of individuals with diabetes with similar medication patterns, and offer valuable insights into the medication characteristics of individuals with diabetes across different age groups. Despite potential biases and methodological issues, our study presents valuable insights into the medication management of diabetes and provides a foundation for future research on personalized medicine and treatment optimization. A deeper description of the methodology is given in the Supplementary file.

## 3. Results

In the following, we present the results of our analysis focusing on the use of medications in the treatment of T2DM. In addition, we consider age groups of individuals with diabetes and identify groups of patients treated in a similar manner. We started from the patients’ medical history and used different databases to identify the ATC code of the medications prescribed to each individual with diabetes after screening.

### 3.1. Frequency of prescribed drugs for different age groups

**Figure 2:**
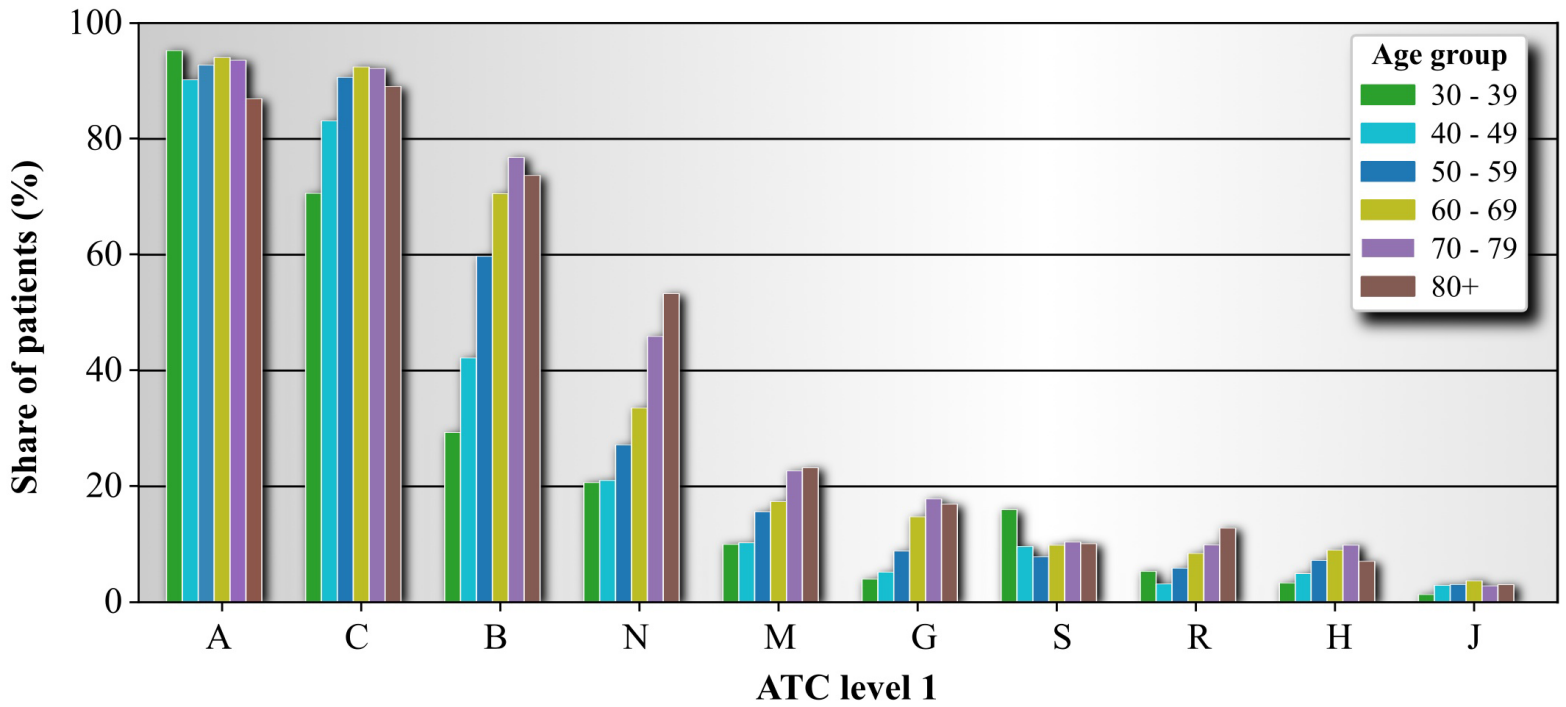
Medication usage frequency based on ATC LV1 for individual age groups. ATC classifiers are organized from the most frequent to the last frequent. Shown are only 10 most used ATC LV1 medications. The ATC level 1 names are Alimentary tract and metabolism (A), Cardiovascular system (C), Blood and blood forming organs (B), Nervous system (N), Musculo-skeletal system (M), Genito-urinary system and sex hormones (G), Sensory organs (S), Respiratory system (R), Systemic hormonal preparations, excluding sex hormones and insulins (H), and Antiinfectives for systemic use (J).

In Figure 2 we show the share of individuals with diabetes in each age group that have been prescribed medications which belong to a given ATC LV1. Overall, we see that most commonly medications from the 1st level class A (Alimentary tract and metabolism), followed by the class C (Cardiovascular system), followed by class B (Blood and blood forming organs), followed by class N (Nervous system), Followed by class M (Musculo-skeletal system). The remaining 1st level ATC classes are prescribed to les then 1/5 of the individuals with diabetes. Furthermore, it has been noticed that the ATC LV1 category, which is the most frequently utilized and encompasses medications for diabetes, exhibits a consistently high usage rate across all age groups, albeit with some variation ranging between 90 and 99%. The second most utilized ATC LV1 class, which includes drugs used in treating cardiovascular diseases, is found in over 70% of individuals with diabetes in the youngest age group of individuals with diabetes. Moreover, the proportion of individuals with diabetes using these medications increases in the subsequent two age groups and stabilizes at approximately 92%. The Blood and blood forming organs medication, which is the third most used ATC LV1 class, is relatively rare in the youngest age group compared to the first two most used medication classes. However, the proportion of individuals with diabetes prescribed drugs from this class significantly increases in the subsequent age groups. A similar trend is observed for medications in the ATC LV1 class N. Among the remaining six medication classes, increasing usage with age can be observed in classes M (Musculo-skeletal system), G (Genito-urinary system and sex hormones), R (Respiratory system), and H (Systemic hormonal preparations, excluding sex hormones and insulins). The usage of medications belonging to the ATC LV1 classes S (Sensory organs) and J (Antiinfectives for systemic use) remains relatively stable across age groups.

**Figure 3:**
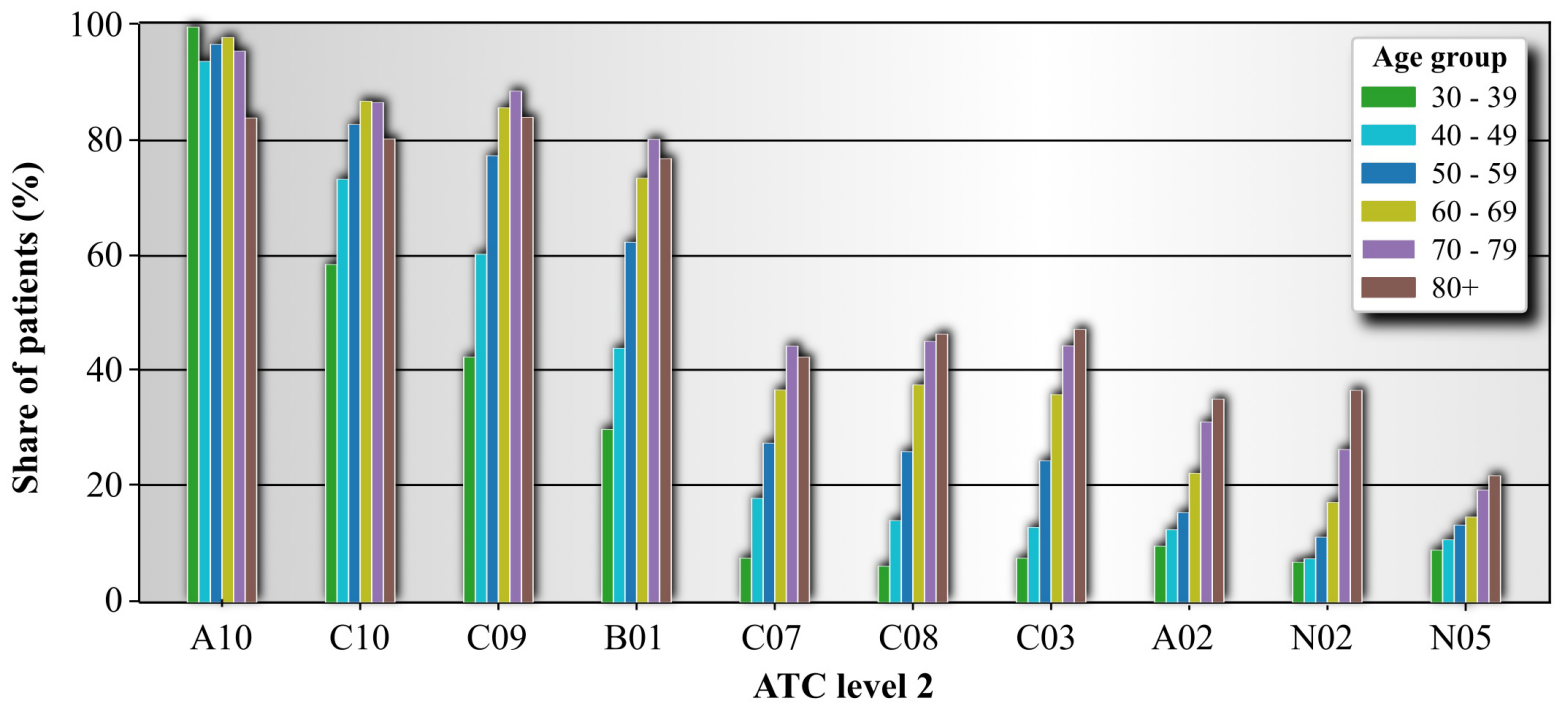
Medication usage frequency based on ATC level 2 for individual age groups. ATC classifiers are organized from the most frequent to the last frequent. Shown are only 10 most used ATC level 2 medication classes. Drugs for acid related disorders (A02), Drugs used in diabetes (A10), Antithrombotic agents (B01), Diuretics (C03), Beta blocking agents (C07), Calcium channel blockers (C08), Agents acting on the renin-angiotensin system (C09), Lipid modifying agents (C10), Analgesics (N02), and Psyholeptics (N05).

To evaluate the frequency of medication use more accurately, we have considered the medication code one level deeper, specifically the ATC LV2 of the prescribed medications. Similarly, we have listed the 10 most used ATC LV2 classes. The most frequently used subgroup of medications among individuals with diabetes with T2DM are those labeled as A10 (Drugs used in diabetes). This result is expected. The second most used medication type based on the ATC LV2 is C10 (Lipid modifying agents), followed by C09 (Agents acting on the renin-angiotensin system) and B01 (Antithrombotic agents). These four subgroups of medications are found in more than 60% of patients over 50 years of age. Continuing, we find the subgroups C07 (Beta-blocking agents), C08 (Calcium channel blockers), and C03 (Diuretics), which are prescribed to over 40% of patients over 70 years of age. Lastly, we find the medication subgroups A02 (Drugs for acid-related disorders), N02 (Analgesics), and N05 (Psycholeptics), which are used by around 20% of patients over 70 years of age. For the medications shown in Figure 3, we see that the prevalence of medication use among individuals with diabetes generally increases with age. However, two exceptions are noted. First, in the oldest age group, the use of the first 5 most frequently used medications decreases. Second, medication subgroup A10 is the only one whose use does not increase monotonically with age.

**Figure 4:**
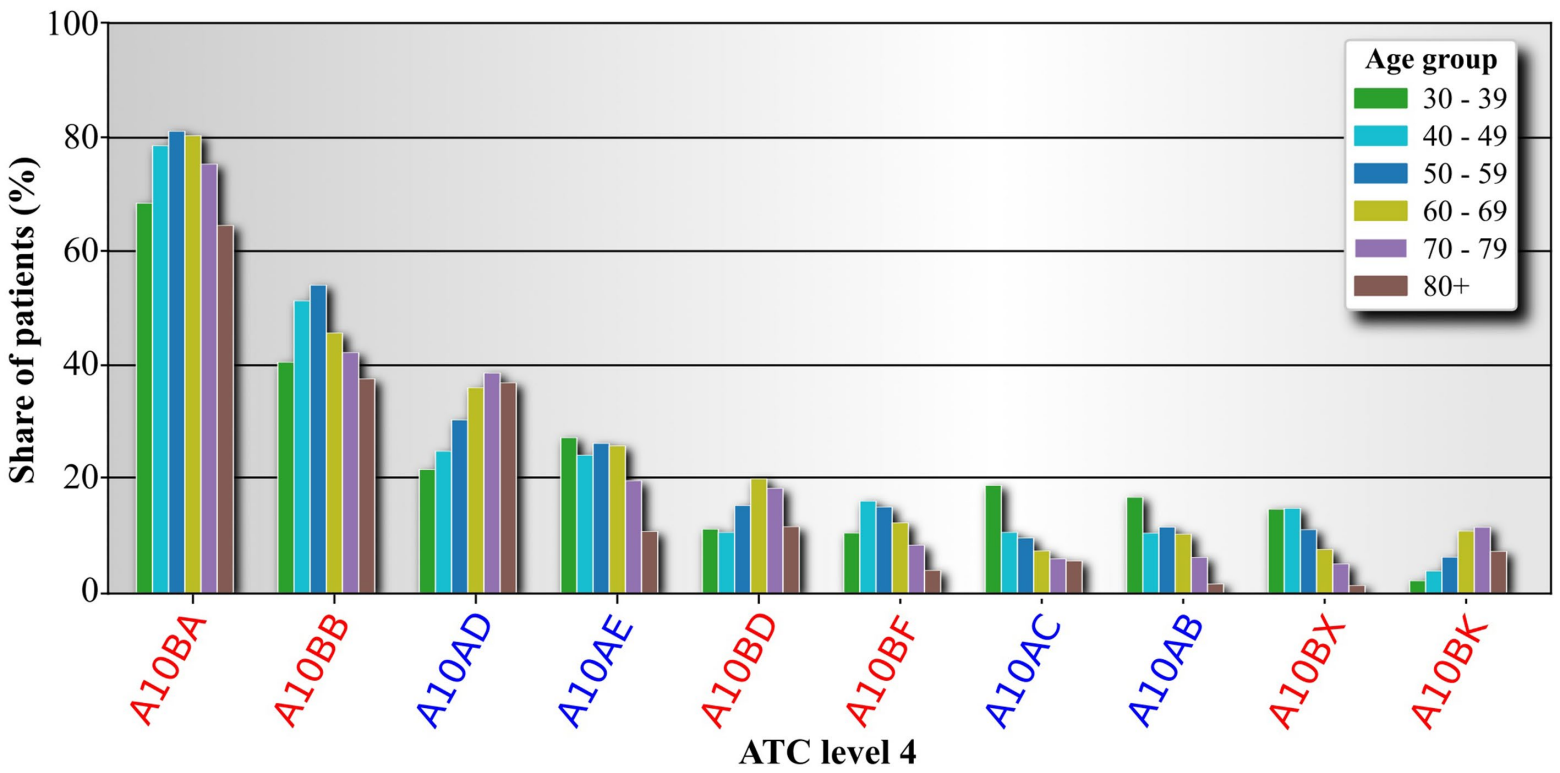
Analysis of drugs belonging to the class Drugs used in diabetes (ATC LV2 A10). Drugs are analyzed on the 4th ATC level. The 3rd level is color coded on the labels. Labels of drug classes originating from the 3rd ATC level A10A are colored blue, and label originating from the 3rd ATC level A10B are colored red. The label names are abbreviations for Biguanides (A10BA), Sulfonylureas (A10BB), Combinations of oral blood glucose lowering drugs (A10BD), Alpha glucosidase inhibitors (A10BF), Other blood glucose lowering drugs, excl. Insulins (A10BX), Sodium-glucose co-transporter 2 (SGLT2) inhibitors (A10BK), intermediate- or long-acting combined with fast-acting (A10AD), long-acting (A10AE), intermediate-acting (A10AC), and fast-acting (A10AB).

In the following sections, we provide a more detailed breakdown of the ATC LV2 class A10 medications used in individuals with diabetes according to their respective age groups. Since there are only two ATC LV3 classes within the A10 medication branch, A10A and A10B, we instead focus on examining the ATC LV4 drugs of the A10 medication branch. The results are presented in Figure 4. The most used drugs across all age groups are the ATC LV4 drugs A10BA (biguanides), followed by the A10BB branch (sulfonylureas). The usage of these two drug branches increases until the 50–59 age group and then begins to decline. We observe an almost monotonic increase in the usage of drugs A10AD (insulins and analogues for injection, intermediate- or long-acting in combination with fast-acting) and A10BK (sodium-glucose co-transporter 2 (SGLT2) inhibitors), which were only introduced in 2014 (Date of first approval: May 22, 2014) (49). On the other hand, the usage of other drug classes in the corresponding 4th ATC tier exhibits a decreasing trend across the age groups.

In continuation we analyze patters in medication treatments among individuals with diabetes.

### 3.2. Diabetic treatment profiles

**Figure 5:**
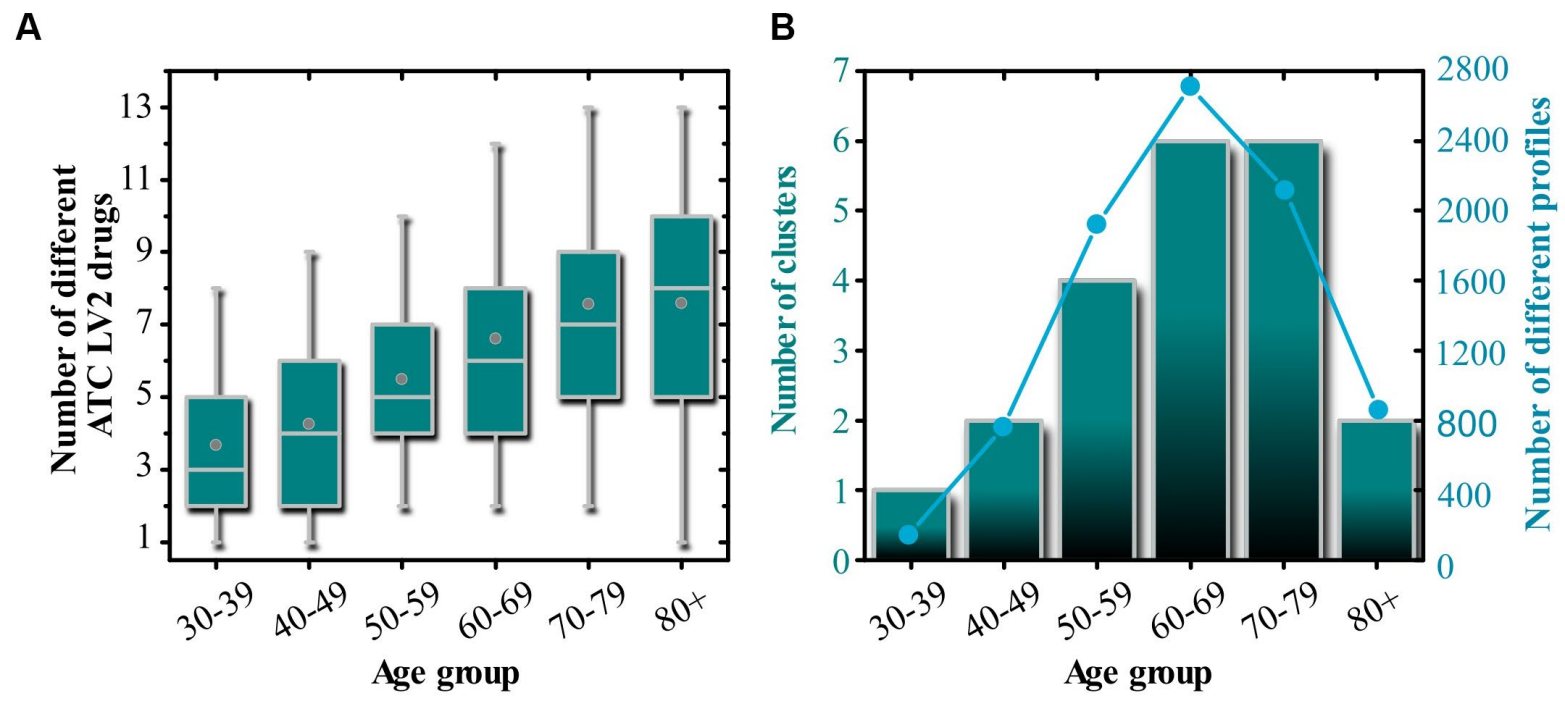
Global profile features. **(A)** Shows box chart diagrams related to the number of different ATC LV2 drug prescriptions in each age group. In panel **(B)**, we display the number of extracted clusters based on the drug prescription profiles within individual age groups. The light blue line in panel **(B)** corresponds to the number of different profiles within a given age group. In panel **(A)**, the boxes in the boxplot enclose the middle 50% of the data. The vertical line inside the box represents the median of the data, while the lower and upper edges of the box represent the first and third quartiles, respectively. The whiskers extend from the box to the 5 and 95% values of the data, but not including outliers. Circles inside the boxes represent the mean value.

We have now shifted our focus to detecting and describing homogeneous groups of individuals with diabetes based on their prescribed medication profiles within 10-year age groups (i.e., 30–39, 40–49, etc.). We employed hierarchical clustering to identify such similar patients, holding the similarity threshold constant at 16. A higher threshold would result in a lower number of extracted clusters, while a lower threshold would yield more clusters. A detailed description of how the medication profiles were created and how hierarchical clustering was performed can be found in the Material and Methods section. Overall, we found that the average number of different drugs prescribed to a patient, according to the 2nd ATC level, increases with age groups. Thus, it is not surprising that the number of clusters also increases with age. A higher variability in drug consumption is expected to influence the extracted groups of individuals with diabetes with similar drug prescriptions. The results are presented in Figure 5.

The results of the patient profile analysis presented in Figure 5 are consistent with those of Figures 2–4. In general, we observe an increasing trend in the number of different ATC LV2 drugs with increasing age groups (see Figure 5A). Figure 5B displays how the number of clusters changes along with the number of individuals with diabetes in each age group. We find that the number of patients increases from the youngest age group (30–39 years) to the age group (60–69 years), after which a decrease is observed. Similarly, we observe an increase in the number of clusters from the youngest age group to the age group (60–69 years), with the number remaining constant in another age group, and decreasing drastically in the oldest age group.

**Figure 6:**
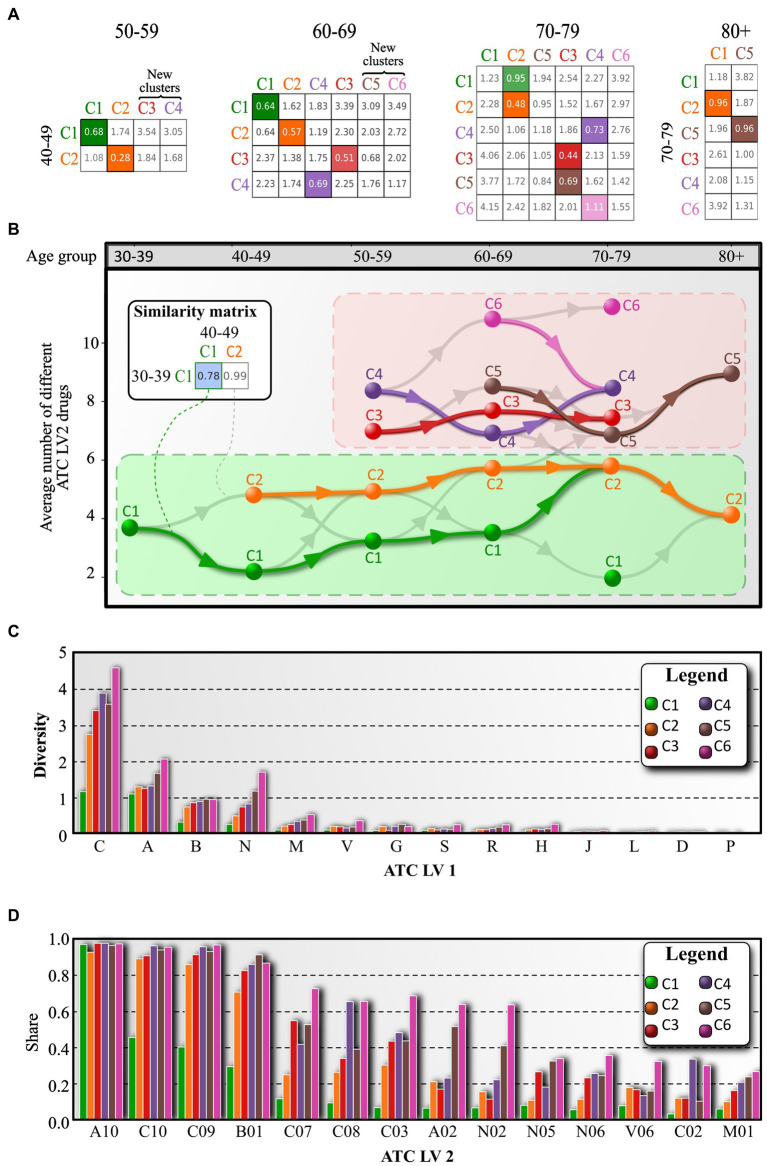
Tracing of cluster-specific drug profiles across adjacent age groups. **(A)** Distance matrix between average drug profiles of cluster of adjacent age groups. Cells of the matrix are colored based on the most similar cluster in the previous age group. **(B)** According to the distance matrix, clusters are color coded with respect to the color of the most similar cluster in the previous age group. However, each cluster can have only one output link. If a cluster appears that cannot be linked, it is assigned a new color. **(C)** The average number of different ATC LV2 medications of the corresponding ATC LV1 branch. **(D)** The proportion of individuals with diabetes prescribed a particular ATC LV2 drug. The bars are color coded according to the cluster for which the data were averaged. In addition, panel **(D)** lists only the 14 most common ATC LV2 drugs.

As we proceed, we extend our analysis to individual clusters, which are groups of individuals with diabetes that share some degree of similarity. As described in the Materials and Methods section, our goal at this stage is to assess whether two clusters of patients in adjacent age groups can be considered similar. To do this, we calculate which pair of clusters in adjacent age groups has the highest degree of similarity, treating the two most similar clusters as a particular drug profile type that occurs in different age groups. To accomplish this, we first calculate the distance matrix between the average drug profiles of clusters in two adjacent age groups. Using this matrix, we can then track the drug-specific profiles across different age groups. The results of this analysis are shown in Figure 6.

The basis for tracking cluster-specific profiles across age groups is the distance matrix (see Figure 6A). Initially, clusters are labeled based on the average number of different ATC LV2 drugs, with the cluster with the lowest number labeled C1, the second highest labeled C2, and so on. The distance matrix contains the distance measure between a given cluster in one age group and a given cluster in the neighboring age group. The pair of clusters with the highest degree of similarity (i.e., the lowest distance) between the two age groups is considered a similar drug profile type, and the corresponding profile in the older age group inherits the color of the most similar profile in the younger age group. The color of a cluster indicates that the individuals with diabetes are on a similar spectrum of prescribed medicines. We create a directed edge between a pair of clusters that have the highest degree of similarity, and we also add the second most similar path as a directed gray edge to the image shown in Figure 6B.

The results in Figure 6B reveal an important feature of the system, with two groups of clusters emerging. The first group (clusters C1 and C2) is characterized by a relatively low average number of different ATC level 2 drugs, and the profiles appear to evolve in a stable manner, without significant changes (i.e., the orange course does not fall below the green course). In contrast, the second group of clusters is much more dynamic and diverse, with higher average numbers of different ATC LV2 medications, and the location of the individual profile types is less stable. This group includes clusters C3, C4, C5, and C6. And interestingly, the two groups are not directly accessible if we consider only the most similar connections.

The results presented in Figure 6C provide information on the drug prescription diversity for each cluster. The average number of different ATC LV2 drugs from a given ATC LV1 branch prescribed to an average patient is shown. For instance, an average patient from cluster C1 is prescribed approximately 1 drug from ATC LV1 branch A (Alimentary tract and metabolism) and 1 drug from ATC LV1 branch C (Cardiovascular system), while an average patient belonging to cluster C2 takes 2.5 different medications from ATC LV1 branch C. This information sheds light on the diversity of medication spectra in patients and suggests that individuals with diabetes require different treatment strategies based on their individual needs. Figure 6C also shows that the medication diversity increases with age, with clusters belonging to older age groups having greater diversity compared to those belonging to younger age groups. We also note that the diversity of medication prescriptions for ATC LV1 branch C is highly dispersed across clusters, with values ranging from 1.1 to 4.6. This also suggests that individuals with diabetes are highly heterogeneous in terms of their cardiovascular condition. Furthermore, the prescription frequency of ATC LV2 drugs is examined in Figure 6D. The drugs from ATC LV2 branch A10 (Drugs used in diabetes) are prescribed to almost 100% of patients in all clusters, while other ATC LV2 drugs are not prescribed as frequently by individuals with diabetes in cluster C1. Clusters belonging to older age groups have a relatively high prescription frequency for an increasing number of drugs. The distribution of measured serum glucose, cholesterol, and triglycerides is also comparable between drug-specific clusters, as shown in the Supplementary file, but individuals with diabetes require different ranges of drugs to achieve this. For a more detailed description of the range of drug prescriptions for each cluster, considering the age groups of the individuals with diabetes, refer to the Supplementary file.

**Figure 7:**
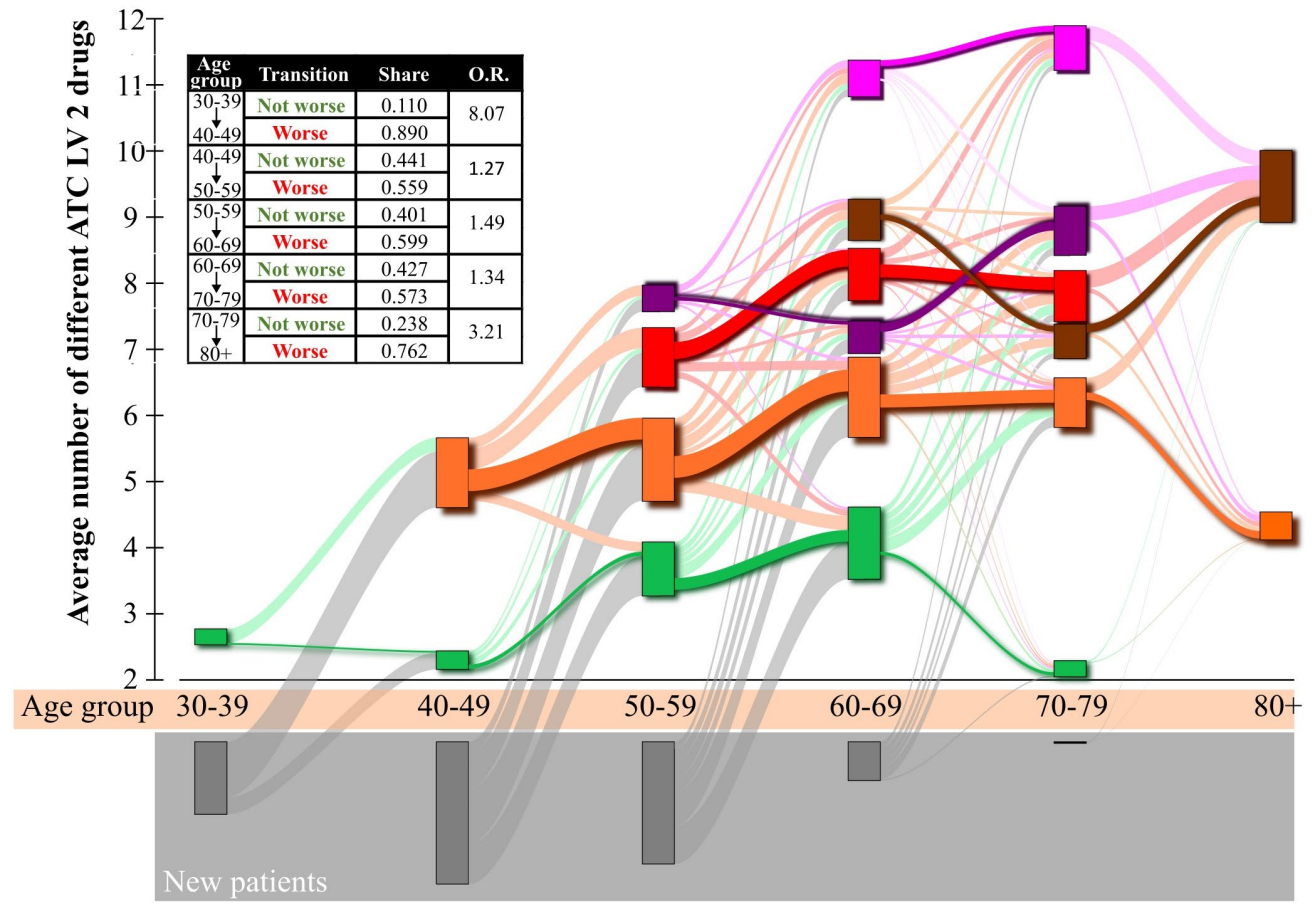
Sankey diagram of patient flows between clusters in adjacent age groups. Individual boxes are color-coded based on the drug-specific cluster profile. The boxes are arranged according to the embedded age group of individuals with diabetes (x-axis) and their average number of different ATC LV2 drugs (y-axis). The size of the boxes is proportional to the number of embedded individuals with diabetes. The width of the lines connecting two boxes is proportional to the number of individuals with diabetes transitioning from one box to another. The gray boxes at the bottom of the diagram mark newly introduced individuals with diabetes in a certain age group.

Finally, the analysis of the patient flow is shown in Figure 7. We visualize this flow chart because one of the criteria for including a patient in our analysis was that he or she had been monitored for more than 10 years. Since we have 10-year age groups, a patient can be found in at least two age groups, and patient tacking is possible. Figure 7 clearly shows that as individuals with diabetes age, in most cases they move from a “healthier” group with fewer medications to a group with more complications and more medications. To quantify this, we calculated the proportion of individuals with diabetes who stayed in the same group or transitioned to a “healthier group” when they moved to an older age group. This group of people was designated as Not Worse. At the same time, we also calculated the proportion of remaining individuals with diabetes who regressed after moving to the older age group in the sense that they were distributed in clusters that were “less healthy” or as we labeled them “Worse.” Overall, we find that the odd ratio (O.R.) regression is very favorable in the youngest and oldest age groups. In the other age groups, there is also a higher O.R. for individuals with diabetes transitioning into clusters with a broader range of prescription medications. It is also interesting to note that the homogeneity of the profiles follows a U-shaped curve. Namely, in the youngest age group and the oldest age group, the number of different profiles is relatively small. The number of different profiles increases up to the age of 60–69. From this age group on, the number of clusters remains the same for another age group and decreases sharply thereafter. It is also interesting to observe that the location of all clusters in relation to the average number of different ATC LV2 drugs increases up to the age group of 60–69. This is also the age group where the number of new individuals with diabetes sharply decreases. We also note that in older age groups, the outflow of individuals with diabetes is much lower than the inflow of individuals with diabetes. While this is not necessarily indicative of patient mortality rates, there is certainly a correlation. After the age group 60–69, the influx of new individuals with diabetes sharply decreases. New individuals with diabetes begin to represent only a minority of individuals with diabetes within individual clusters. Most individuals with diabetes are from younger age groups. As a result, a smaller inflow of new individuals with diabetes and outflow of individuals with diabetes from younger age groups results in smaller clusters. Interestingly, some clusters do not significantly increase the average number of different ATC LV2 drugs, and in some cases, they even consume fewer medications. This could indicate that individuals with diabetes who remain in the age group did not use that many medications, or that the number of different medications needed to be restricted due to other reasons. For example, the evaluation of glomerular filtration rate (eGFR) is a measure of how well the kidneys are functioning and is used to assess kidney function. A low eGFR indicates impaired kidney function, and as can be observed in the Supplementary file, we can see that eGFR values begin to decline in all clusters with increasing age groups. However, the decline is not uniform. We observe that for individuals with diabetes, belonging to the cluster with the highest average number of ATC LV2 drugs, the eGFR values are much lower compared to the eGFR values for the cluster of individuals with diabetes, which consume the lowest number of different ATC LV2 medication (see Supplementary file section 2). This is in line with the results and thus supports the accuracy of the methodology in detecting patient profiles that have a similar spectrum of medicines.

## 4. Discussion

Diabetes is a major global health issue affecting millions of people worldwide. The management of diabetes involves the use of various medications, which are often prescribed based on individual patient profiles. However, understanding the patterns of medication use and their association with clinical outcomes remains a challenge. In this study, we developed a novel natural language processing (NLP) algorithm to extract information related to prescribed medications from medical records of diabetic individuals with diabetes and their laboratory test results. One main problem we have been able to solve is the use of simple text files created by physicians and the extraction of all for us relevant information from the files in a chronologically structured manner. It is important to point out that the records created by physicians are not linked to other databases or registry. As a results, we had to build a language model capable of extracting all the information’s. Therefore, this solution can autonomously analyze Slovenian plain text files in a structured and organized way, allow additional analyses if needed. In this manuscript, we have used the information and develop a methodology that allowed us to create patient-specific profiles and identify clusters of individuals with diabetes with similar medication patterns, offering valuable insights into the medication characteristics of individuals with diabetes across different age groups and evaluate key differences between the extracted profiles.

Our findings revealed that diabetic individuals with diabetes were most likely to receive medications belonging to ATC level 1 class A (Alimentary tract and metabolism), followed by class C (Cardiovascular system), and then class B (Blood and blood-forming organs). This is consistent with the fact that diabetes is primarily a metabolic disorder and that diabetic individuals with diabetes are at an increased risk for cardiovascular complications (50). Interestingly, our results showed that the use of medications in class B was more prominent in older age groups, suggesting that the management of diabetes may become more complex as individuals with diabetes age.

In terms of diabetes-specific medications, we found that biguanides and sulfonylureas were the most prescribed drugs. This finding is in line with existing literature, which reports that metformin (a biguanide) is often the first-line treatment for T2DM, followed by sulfonylureas as an additional therapy when needed (51). We also observed an almost monotonically increasing trend in the use of A10AD (insulins) and A10BK (SGLT2 inhibitors). This observation might reflect the growing popularity of these drug classes in diabetes management due to their demonstrated efficacy and safety profiles (52, 53). Interestingly, some drugs originally developed for one indication have found new therapeutic applications. For instance, sodium-glucose cotransporter-2 (SGLT-2) inhibitors, initially used as antidiabetic drugs, have demonstrated significant benefits in cardiovascular and renal diseases. Drugs like empagliflozin and canagliflozin, first approved for managing blood glucose levels in Type 2 diabetes patients, have subsequently received approval for heart failure and chronic kidney disease treatment, respectively (54, 55). Under the Anatomical Therapeutic Chemical (ATC) classification system, each drug is assigned a unique code based on the main condition it treats. For instance, empagliflozin carries the ATC code A10BX09, which denotes its primary use in diabetes. However, this code may not fully capture the diverse therapeutic potential of the drug, given its extended indications for heart failure and kidney disease.

Our study also revealed an increasing trend in the number of different ATC level 2 drugs prescribed as individuals with diabetes aged, with the highest number of individuals with diabetes found in the age groups 50–59 and 60–69. These results align with previous research indicating that the prevalence of diabetes and its complications increases with age (56). Moreover, we found that the population of female individuals with diabetes reached its peak of prescribed medications a decade later than male individuals with diabetes, which is consistent with our previous findings on age-related changes in lipid and glucose levels associated with drug use and mortality (57). This observation underscores the importance of effective diabetes management, as diabetes-related mortality remains a significant public health (58, 59).

In our observation, we found that although individuals with diabetes were being treated with various medication strategies, serum glucose and cholesterol values mostly did not show significant differences among the different treatment groups (see Supplementary file). However, when we consider serum triglyceride values and estimated glomerular filtration rate (eGFR) values, we observe a pattern. For serum triglyceride values, we see that cluster C1, which is the cluster that, on average, consumes the smallest number of different medications, consistently exhibits a different distribution compared to the average individual with diabetes. It also has the lowest median value of triglycerides. Regarding eGFR values, we observe that clusters that, on average, consume more different medications are characterized by lower eGFR values. This discovery may clarify why individuals with diabetes are being transferred from the cluster with a high number of different ATC LV2 drugs to the group with the fewest number of different ATC LV2 drugs. Namely, if a patient’s eGFR value is too low, it may be recommended to abandon some medications. The number of clusters found to have statistically different distributions is the highest for these features.

One of the major strengths of this study is the development and implementation of a novel NLP algorithm, which can efficiently extract medication information from the EMR of individuals with diabetes. The language model is trained on specific medical records, which enables it to process large amounts of textual data, providing valuable insights into medication characteristics and usage patterns. Another significant strength is the comprehensive analysis of EMR, which allowed for the identification of medication clusters and the flow of individuals with diabetes between clusters in different age groups. This detailed analysis offers insights into medication usage patterns and their potential implications for diabetes management and patient care.

However, several limitations should be noted. It would be worthwhile to integrate our data into different diabetes clusters, such as those reported by Ahlqvist et al. (13). The current capabilities of our language model for information extraction are currently not capable of accurately extracting all the needed information’s, for such a task. However, we are developing a more powerful language model capable to extract a much broader spectrum of information’s from EMRs, which would allow us integrating results for the diabetes clusters reported by Ahlqvist et al. (13). Additionally, there is the possibility of biases in the data, such as selection bias or information bias. This could influence the results. For instance, the EMR may not accurately capture all medications prescribed to individuals with diabetes, or they may have missing or incomplete information. Additionally, methodological issues, such as clustering approach, distance metrics, and linkage methods, could affect the interpretation of medication usage patterns and their clinical implications. Future research could explore alternative clustering algorithms and testing different distance metrics and linkage methods to obtain more robust and reliable findings that can inform clinical practice and personalized treatment strategies for individuals with diabetes.

In conclusion, future research in the field of diabetes medication management could benefit from further refining the NLP algorithm, incorporating additional data sources, and examining the relationship between medication regimens and patient outcomes in more detail. These efforts could lead to more accurate and comprehensive patient profiles, a better understanding of medication usage patterns and their clinical implications, and the development of more personalized treatment strategies that optimize therapeutic benefits while minimizing adverse effects. Despite these limitations, our study presents valuable insights into the medication management of diabetes and provides a foundation for future research on personalized medicine and treatment optimization.

## Data availability statement

The data analyzed in this study is subject to the following licenses/restrictions: The Department of Endocrinology and Diabetology, University Medical Center, provided data supporting the findings of this study. Restrictions apply to the availability of the raw data used in this study; therefore, the data are not publicly available. However, the derived data supporting the findings of this study are available upon request from the corresponding authors. Requests to access these datasets should be directed to matej.zavrsnik1@gmail.com; rene.markovic@um.si.

## Author contributions

MM set out the project, the main conceptual ideas, and proof outline. MM, RM, VG, and MZ contributed to the design of the research. MM, RM, and VG wrote the manuscript with assistance from MZ, PK, and MP. The dataset was provided by JZ, TZ, and MZ. Data analysis was performed by RM who also visualized the results. RM, VG, TZ, HB, PK, MP, MM, MZ, and JZ provided critical feedback and helped shape the research, analysis, and manuscript. All authors contributed to the article and approved the submitted version.

## Funding

This research was supported by the Slovenian Research Agency (Javna agencija za raziskovalno dejavnost RS) (Grant Nos. P1-0403, J1-2457, N1-0232, J3-3077, J5-3114, P1-0055, and I0-0029).

## Conflict of interest

The authors declare that the research was conducted in the absence of any commercial or financial relationships that could be construed as a potential conflict of interest.

## Publisher’s note

All claims expressed in this article are solely those of the authors and do not necessarily represent those of their affiliated organizations, or those of the publisher, the editors and the reviewers. Any product that may be evaluated in this article, or claim that may be made by its manufacturer, is not guaranteed or endorsed by the publisher.
