# Supplementary material for: Profiling of patients with type 2 diabetes based on medication adherence data
Source: Front Public Health. 2023 Jul 6;11:1209809. doi: 10.3389/fpubh.2023.1209809 (PMC10358769; doi:10.3389/fpubh.2023.1209809)
Supplement: Supplementary file 1 [file Data_Sheet_1.pdf]

Supplementary Material

Profiling of Patients with Type 2 Diabetes based on Medication Adherence Data

Rene Markovič[1,2], Vladimir Grubelnik[2], Tadej Završnik[3,4], Helena Blažun Vošner[5,6,7], Peter Kokol[2], Matjaž Perc[1,7,8,9,10], Marko Marhl[1,4,11], Matej Završnik[12*], Jernej Završnik[1,5,7,13*]

[1] Faculty of Natural Sciences and Mathematics, University of Maribor, Maribor, Slovenia
[2] Faculty of Electrical Engineering and Computer Science, University of Maribor, Maribor, Slovenia
[3] University Clinical Medical Centre Maribor, Maribor, Slovenia
[4] Faculty of Medicine, University of Maribor, 2000 Maribor, Slovenia
[5] Community Healthcare Center Dr. Adolf Drolc Maribor, 2000 Maribor, Slovenia
[6] Faculty of Health and Social Sciences, Slovenj Gradec, Slovenia
[7] Alma Mater Europaea—ECM, Maribor, Slovenia
[8] Complexity Science Hub Vienna, Vienna, Austria
[9] Department of Medical Research, China Medical University Hospital, China Medical University, Taichung, Taiwan
[10] Department of Physics, Kyung Hee University, Dongdaemun-gu, Seoul, Republic of Korea
[11] Faculty of Education, University of Maribor, 2000 Maribor, Slovenia
[12] Department of Endocrinology and Diabetology, University Medical Center Maribor, Maribor
[13] Science and Research Center Koper, 6000 Koper, Slovenia

*** Correspondence:** jernej.zavrsnik@zd-mb.si; matej.zavrsnik@ukc-mb.si

1 Methodology

1.1 Study design and data sources

We obtained the data from the Maribor University Hospital (MUH) data center. The MUH staff provided us with a database of anonymized records of patients treated in the Department of Endocrinology and Diabetology (DED). The patient records were from 1997 to 2020, and the original dataset contained a total of 213,345 records for 20,793 different patients. Although the dataset obtained from the Data Centre was specifically queried for records created in DED, we add new criteria for extracting only patients with T2DM. To do this, we examined up to 10 diagnoses if reported for a record in the dataset. Diagnoses were based on the International Classification of Diseases (ICD-11), although ICD-10 was also used for older records (1). First, we rejected all patients screened under the diagnosis O24 (diabetes mellitus (diabetes) during pregnancy). Next, we selected only records in which the diagnosis E11 (or a diagnosis derived from this branch of diagnosis) was reported as one of up to 10 diagnoses. Diagnosis E11 is T2DM. In addition, because we relied on the medical history text in our analyses, we selected only records in which the medical history text was longer than 100 characters. Finally, we excluded records belonging to patients who had been examined less than 10 times within a 10-year period and patients younger than 30 years. The result is the herein used database consisting of 75,562 records from 3,886 different patients. To clarify the demographics of the final patient population, we also report basic demographic information on the number of patients who had their first screening at DED at a given age. Patients were divided into 10-year age groups (i.e., the 30-39 age group includes patients aged 30 to 39 years, inclusive). In addition, we also report the distribution of time periods during which patients in the corresponding age groups were continuously screened. The

process of patient selection and the corresponding demographic characteristics are shown in Supplementary Figure 1.

A detailed description of the time periods in which patients have been monitored is shown in Supplementary Figure 2. The period during which the patients were monitored is shown in Supplementary Figure 2. Supplementary Figure 2A shows, for each patient, the time interval during which the data from the appointments with the diabetologist were available. The patient's horizontal curve is color coded according to the age group to which the data are assigned. Supplementary Figure 2B also shows two important pieces of information. First, what proportion of patients in each age group are new, meaning that they have not been seen in previous age groups. Second, what proportion of patients in each age group are from previous age groups. For example, we can see that in the first age group (30-39 year) 100% of the patients originate from the age group 30-39 years. The share of newly added patients monotonically declines and is in the age group 80+ years only 1.6%. We also observe, that from the age group 70-79 year on, newly added patients represent the minority or less than 50% of patients within the age group. We can also observe that the population of female patients reaches its peak a decade after the male population reaches its peak.

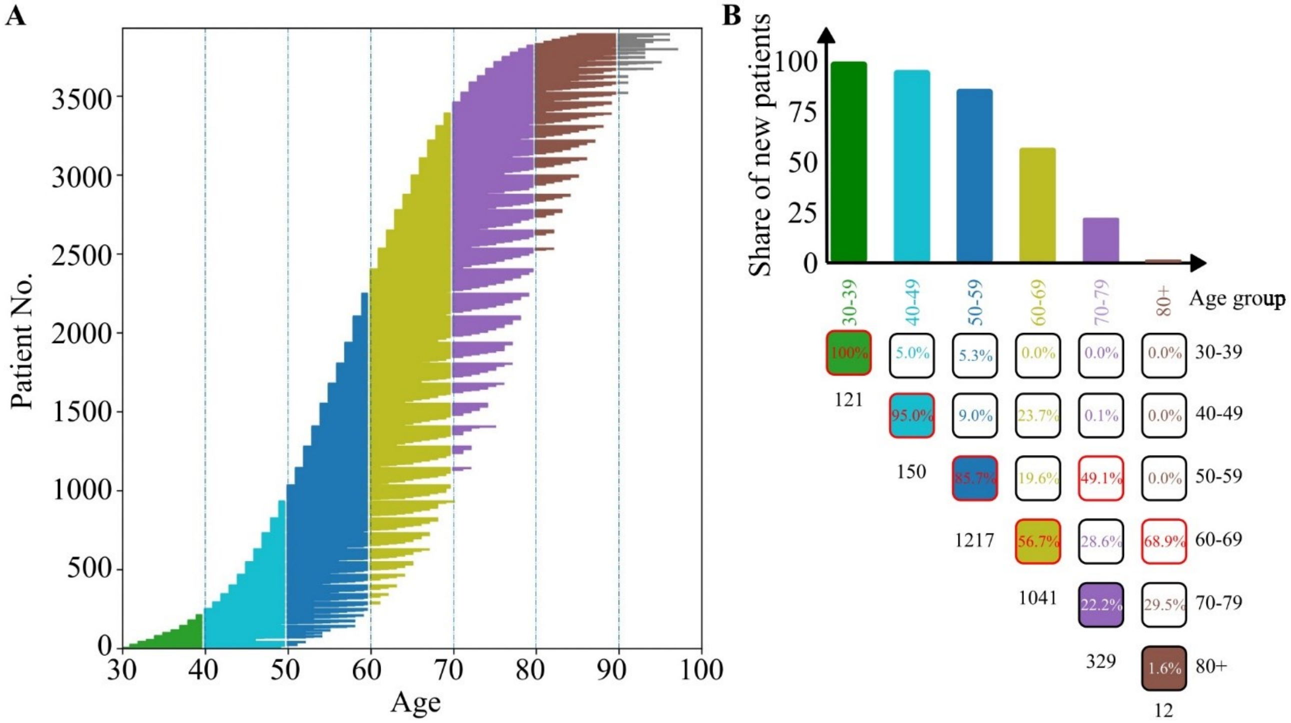

Supplementary Figure 1. Periods of patient monitoring. (A) Initial and final ages for which continuous monitoring data are available for a patient. (B) Composition of age groups based on the starting age of patients within a given age group. The histogram in panel (B) shows the proportion of new patients within a given age group. The matrix below the histogram shows the composition of a given age group in terms of the proportion of patients who belong to a given age group. The sum of the columns in the matrix corresponds to 100%, with the absolute number of patients within an age group indicated below the diagonal element of the matrix.

1.2 Text mining

We placed great emphasis on identifying the medications prescribed to each patient and recorded in the text of the medical record. Thus, for the analysis, we obtained a set of all medications listed in the database of the Central Drug Database or CDD. CDD is the central national reference database for drugs. The database is managed by the Ministry of Health of the Republic of Slovenia, the Public Agency for Medicines and Medical Devices of the Republic of Slovenia, the Slovenian Health Insurance Agency, and the National Institute of Public Health based on an inter-institutional agreement and responsibilities defined by law. Besides the name of a medicine, the corresponding Anatomical Therapeutic Chemical (ATC) code is given. The initial step of our analysis is to collect and combine medical records from our database, which belong to a specific patient. Then, the text combining all medical records for a patient are sorted chronologically from oldest to newest. Going through the entire text, we used regular expressions to normalize the text and search for all medications listed in the CDD. If a medication listed in CDD was found in the text, we saved it and the corresponding ATC code. Lastly, we created a table for each patient, which chronologically listed the prescribed medications. The process is shown in Supplementary Figure 3.

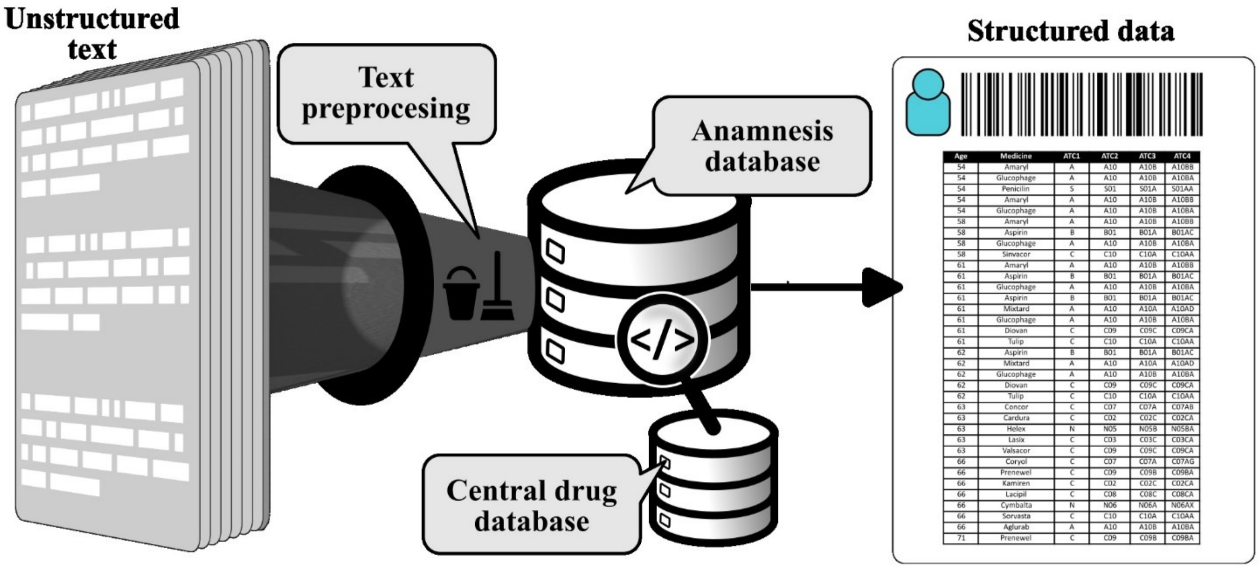

Supplementary Figure 2. Data selection. (A) Patient query pipeline and (B) the number of male and female patients and the time span of the corresponding records by 10-year age groups. Patients were assigned to a specific age group based on age at initial presentation.

This table is used to create patient profiles that reflect the range of medications patients take during their diabetes treatment. More precisely, we focus on the ATC codes rather than the names of the drugs. This is because the ATC classification scheme divides drugs according to the organ or system on which they act, their therapeutic purpose or nature, and the chemical properties of the drug. Therefore, drugs with different names that have the same ATC code have the same active ingredient and the same indications. In addition, the ATC classification system is hierarchical. The first ATC level (ATC LV1) designates the main anatomical group and consists of one letter. The second ATC level (ATC LV2) corresponds to the therapeutic subgroup and consists of two digits. The third level (ATC LV3) denotes the therapeutic/pharmacological subgroup and consists of one letter. In our analyses we will analyze the medication usage features to up to the 4th level (ATC LV4). At this level, the classification system

groups drugs according to their chemical characteristics. It is based on the active ingredient of the drug, which is the substance that is responsible for the drug's therapeutic effect. Each drug at this level is assigned a unique four-letter code that reflects the chemical structure of the active ingredient.

1.3 Definition of a patient profile and cluster extraction

For each patient, we broke down his data by age group. As an example, we have a patient who was treated at ages 45, 46, 49, 50, and 52. We aggregate records obtained by the patient at ages 45, 46, and 49 and use them in the analysis within the 40-49 age group. Records obtained at ages 50 and 52 are aggregated and used in the analysis of the 50-59 age group.

To identify homogeneous commonalities within age groups based on patient medication adherence data, we define a prescription profile for each patient. The profile is created by identifying, within a given age group, which medications the patient has received. Thus, for each medication, we review its ATC LV2 medication list. If the patient has received a drug in a particular ATC group, it is noted. There are 94 possible drugs at the ATC LV2. Consequently, the profile of each patient has 94 dimensions for each age group to which he belongs. Thus, for a single patient, we define a binary vector with 94 values. The individual values of the vector can be 0 if the patient did not receive a specific ATC LV2 drug. For ATC LV2 drugs, on the other hand, which have been prescribed to the patient, the corresponding vector value is set to 1.

Having obtained the profiles, we proceed to compute the distance matrix between any pair of patients within a given age group. For simplicity, we assign the symbol $p_{i,AG}$ to the profile of a patent within a particular age group, where the indices i and AG each correspond to a particular patient within a particular age group. The distance is calculated as a Euclidean distance as follows:

$$d(i,j,ag) = \sum_{k=1}^{94} \sqrt{\left[p_{i,AG}(k) - p_{j,AG}(k)\right]^2}. \quad (1)$$

In equation (1), the distance between the i-th and j-th profiles of any two patients within a given age group is denoted by $d(i, j, ag)$. The sum in Eq. (1) goes through all 94 elements of the profiles, with the k-th element of i-th patient profile denoted as $p_{i,AG}(k)$. The difference between two elements $p_{i,AG}(k) - p_{j,AG}(k)$, can be either 0 or 1. If two patients either received or did not receive a particular ATC LV2 drug, the corresponding difference is 0. On the other hand, if one patient is prescribed a particular ATC LV2 drug but the other patient is not, the corresponding difference is 1. Thus, the highest possible difference between any two profiles is 94. By computing the distances between any two profiles within a given age group, we define the distance matrix $\mathbf{D}(ag)$ of the age group, with its elements labeled as $d(i, j, ag)$. This matrix is used to capture the similarity of profiles and is the basis for extracting homogeneous groups of patients treated in a similar way in an unsupervised manner.

To find the homogenous groups of patients we implemented hierarchical clustering (2,3). More specifically we implemented the ward. At the initial step of this method, every patient represents its own cluster. Next, the algorithm seeks which two patients to combine first, such that afterward the overall sum of squared within-cluster distances is the least increased. This process is continuously

repeated until all groups are not interconnected at a given degree of dissimilarity. Schematically this process is shown in Supplementary Figure 4.

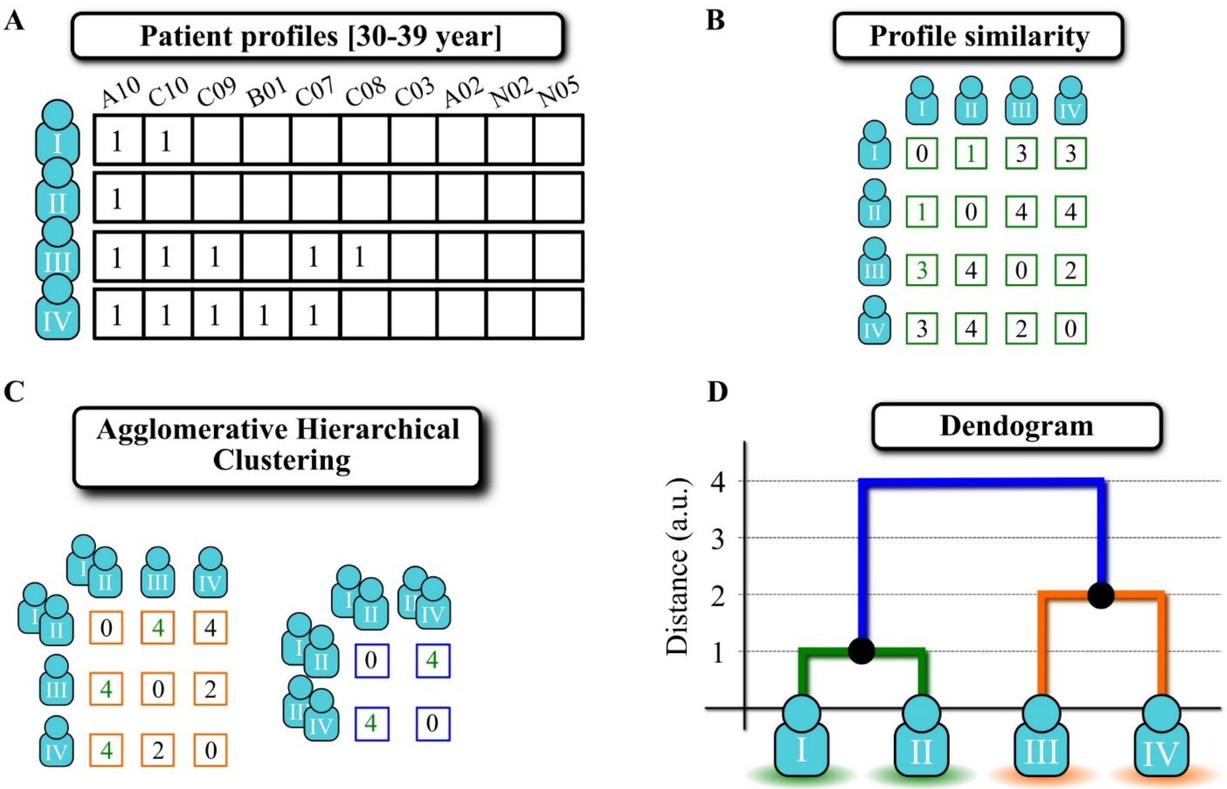

Supplementary Figure 3. Profiling and cluster extraction. (A) Patient-specific profile within a given age group. (B) Distance matrix between corresponding patient profiles. (C) Agglomerative hierarchical clustering of the distance matrix. (D) Visual representation of the extracted groups based on the threshold distance used, D_{TH}.

1.4 Inter age group cluster comparison

Once the clusters within an age group are calculated, we search for the most similar adjacent cluster (adjacent with respect to the age group). For this purpose, we calculate the medication profile for each cluster of patients. The profile is calculated as the average number of different ATC LV2 medications within each ATC LV1 category. For example, if we calculate a value of 1.2 for the ATC LV1 category Medications for Gastrointestinal and Metabolic Diseases, this means that the cluster of patients uses an average of 1.2 different ATC LV2 medications within the ATC LV1 category Medications for Gastrointestinal and Metabolic Diseases. The degree of similarity is the Euclidean distance between the two profiles. Two adjacent clusters that are most similar and therefore have the most common medication usage profile are treated as the same cluster type and are therefore colored the same. If the number of adjacent clusters is greater than the number of clusters in the previous age group, the cluster that is least similar is colored with a new color indicating a new type of medication use profile. In this way, we can not only describe clusters based on the average number of medications received by the corresponding patient group, but also establish a temporal link between two clusters in two adjacent age groups based on the degree of similarity of the medication use profiles. At this point, it should also be mentioned that a patient can be in up to three age groups. However, new patients may appear in

each age group. It is also worth mentioning that a patient who belonged to a certain cluster in one age group may belong to a different cluster in the next age group. This process is shown in Supplementary Figure 5.

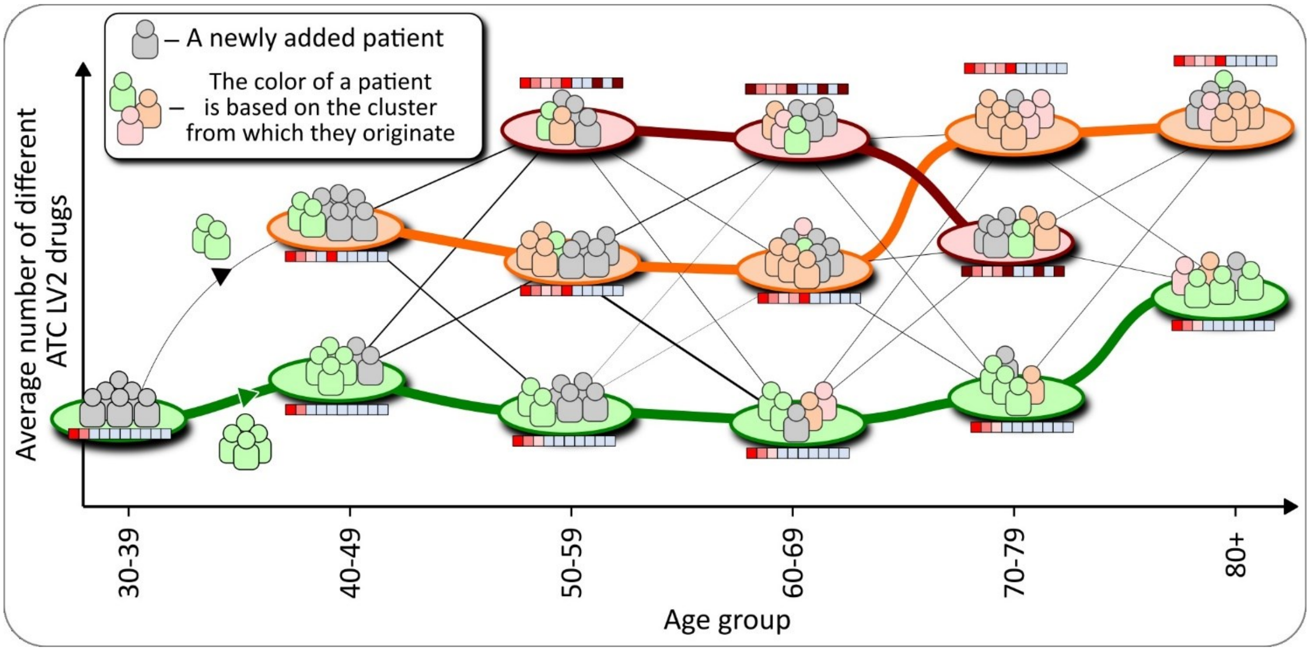

Supplementary Figure 4. Inter-cluster similarity. Individual circles indicate a specific cluster of patients and are located based on the corresponding age group and the average number of different ATC LV2 medications. Upper panel schematically lower panel actual data.

2 Unsupervised patient group detection

In the following section, we delve deeper into the analysis of different age groups. We've created a series of supplementary figures, each representing a specific age group. These figures help us understand the commonalities and differences in medication prescriptions within each age group. Each figure is divided into three parts. The first part, Panel A, is a tree-like diagram, called a dendrogram, which groups patients based on the similarity of their prescribed medications. A dashed line in this diagram shows the level of similarity we used to form these groups. The second part, Panel B, shows the variety of main medication categories (known as ATC Level 1) that an average patient in each group is prescribed. The third part, Panel C, shows the percentage of patients in each group who are prescribed a specific type of medication (known as ATC Level 2). In all parts, we've focused on the 10 most commonly prescribed medications within each medication category. This approach helps us understand the most common medication patterns within each age group.

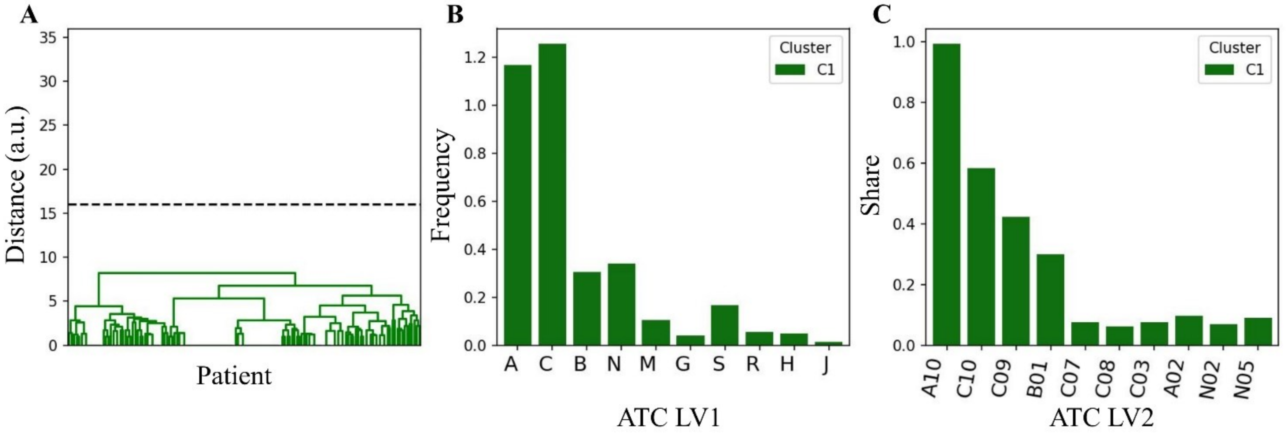

Supplementary Figure 6. Dendrogram and cluster specific medication features for the age group 30-39. (A) Dendrogram, with a horizontal dashed line indicating the distance threshold for grouping patients into clusters. (B) Frequency of ATC LV1 drug occurrence. (C) Share of patients, which are being prescribed a specific ATC LV2 medication. Bars are color coded based on the cluster, they describe.

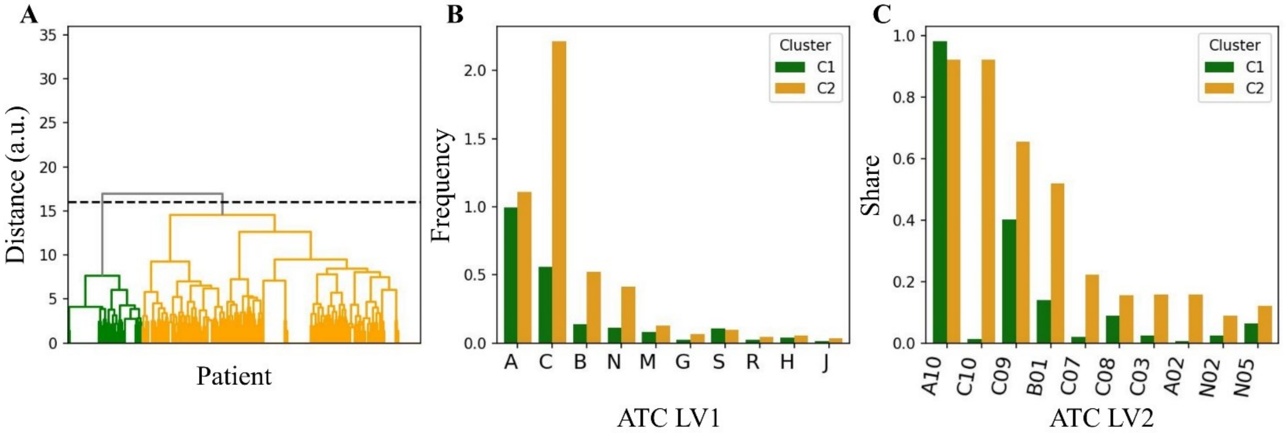

Supplementary Figure 7. Dendrogram and cluster specific medication features for the age group 40-49. (A) Dendrogram, with a horizontal dashed line indicating the distance threshold for grouping patients into clusters. (B) Frequency of ATC LV1 drug occurrence. (C) Share of patients, which are being prescribed a specific ATC LV2 medication. Bars are color coded based on the cluster, they describe.

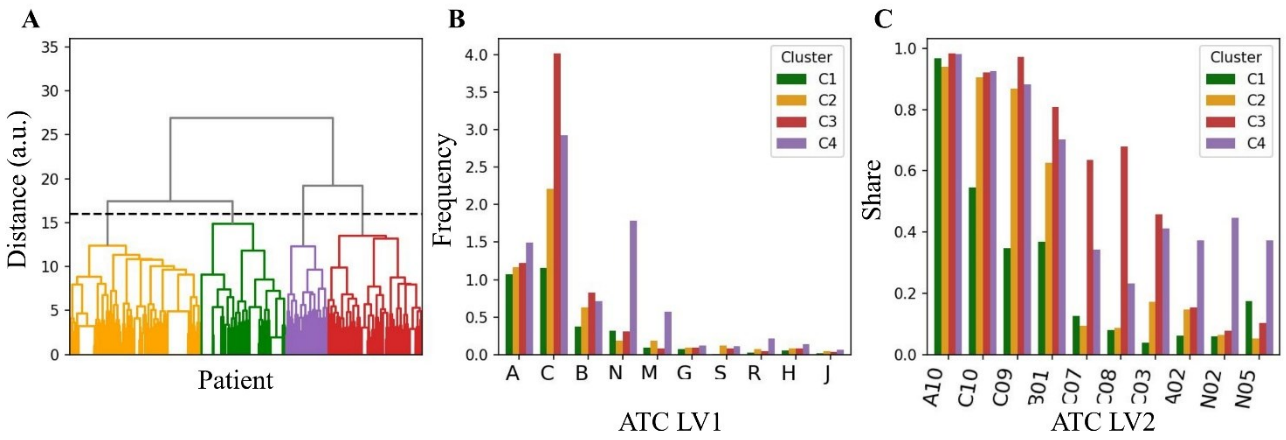

Supplementary Figure 8. Dendrogram and cluster specific medication features for the age group 50-59. (A) Dendrogram, with a horizontal dashed line indicating the distance threshold for grouping patients into clusters. (B) Frequency of ATC LV1 drug occurrence. (C) Share of patients, which are being prescribed a specific ATC LV2 medication. Bars are color coded based on the cluster, they describe.

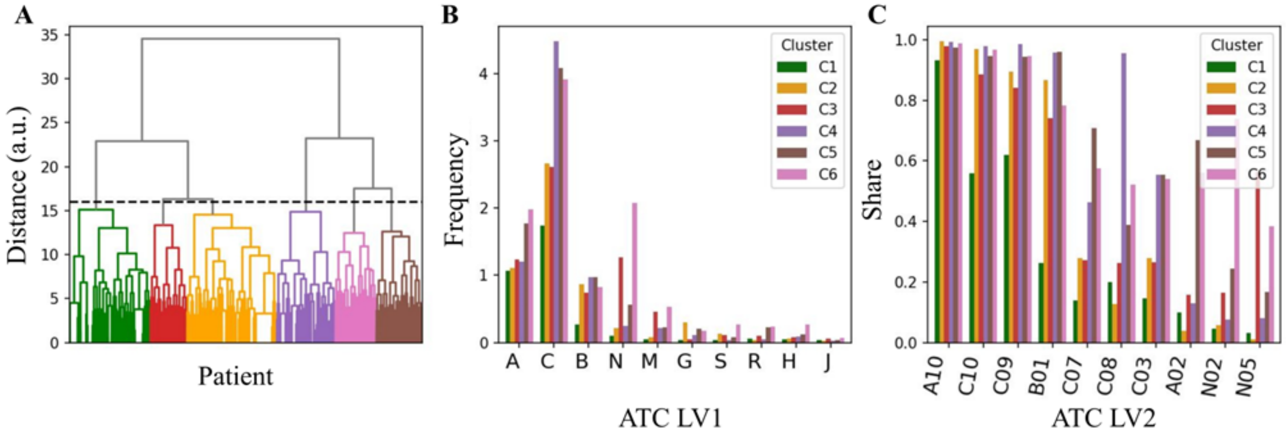

Supplementary Figure 9. Dendrogram and cluster specific medication features for the age group 60-69. (A) Dendrogram, with a horizontal dashed line indicating the distance threshold for grouping patients into clusters. (B) Frequency of ATC LV1 drug occurrence. (C) Share of patients, which are being prescribed a specific ATC LV2 medication. Bars are color coded based on the cluster, they describe.

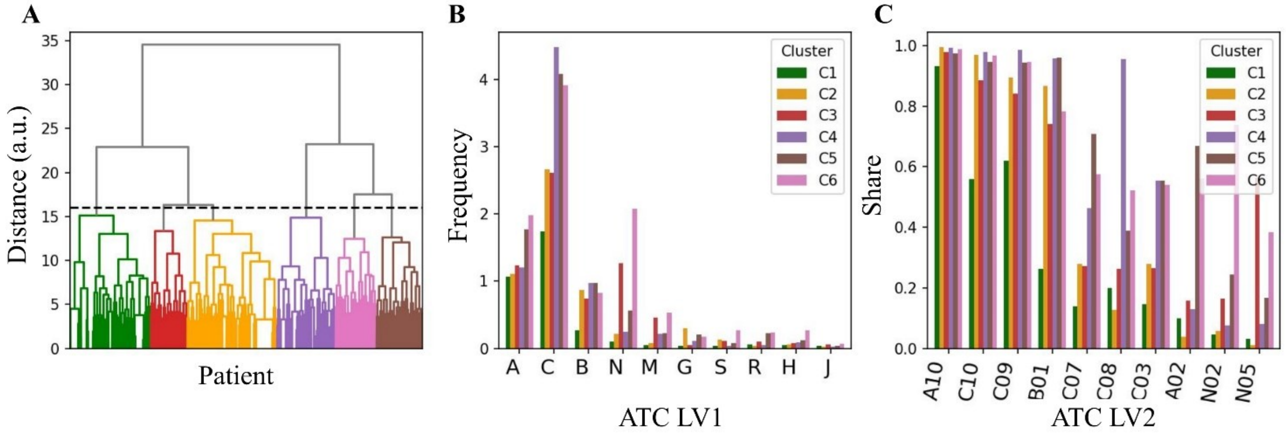

Supplementary Figure 10. Dendrogram and cluster specific medication features for the age group 70-79. (A) Dendrogram, with a horizontal dashed line indicating the distance threshold for grouping patients into clusters. (B) Frequency of ATC LV1 drug occurrence. (C) Share of patients, which are being prescribed a specific ATC LV2 medication. Bars are color coded based on the cluster, they describe.

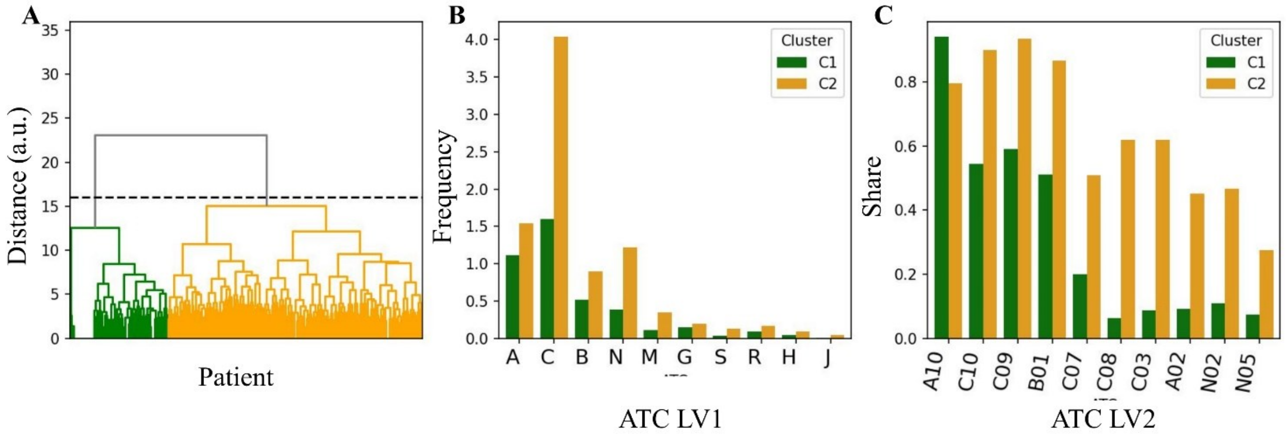

Supplementary Figure 11. Dendrogram and cluster specific medication features for the age group 80+. (A) Dendrogram, with a horizontal dashed line indicating the distance threshold for grouping patients into clusters. (B) Frequency of ATC LV1 drug occurrence. (C) Share of patients, which are being prescribed a specific ATC LV2 medication. Bars are color coded based on the cluster, they describe.

3 Hematological features of diabetic patients in different age groups

Below are box plots of hematological test results for serum glucose, serum cholesterol, serum triglycerides, and estimated glomerular filtration eate (eGFR). Distributions of individual clusters within each age group were compared to the distribution of the entire population in the corresponding age group to test if the sub-population with a specific medication profile has a significantly different distribution compared to the population. We applied the Mann-Whitney U test, a non-parametric statistical test used to compare the distributions of two independent groups. We rejected the null hypothesis (that there is no difference in medians or rank sums between the two groups) for p-values below 0.001. The results are presented in Supplementary Figure 12 to Supplementary Figure 15.

In most cases, we observe one or two clusters within an age group that significantly exhibit different distributions of serum values. This is especially true for serum glucose and cholesterol values. However, when we consider serum triglyceride values and eGFR values, we observe a pattern. For triglyceride values, we see that cluster C1, which is the cluster that, on average, consumes the smallest number of different medications, consistently exhibits a different distribution compared to other clusters. It also has the lowest median value. Regarding eGFR values, we observe that clusters that, on average, consume more different medications are characterized by lower eGFR values. For these features, the number of clusters found to have statistically different distributions is the highest.

SERUM GLUCOSE (mmol/L)

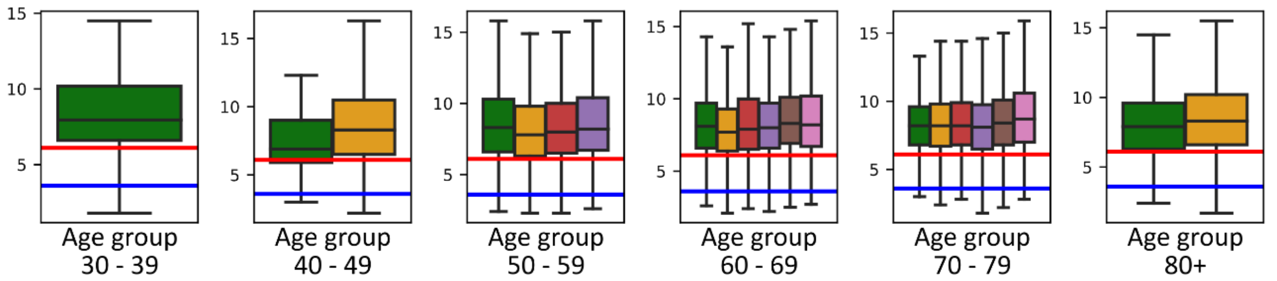

Age group 40 - 49

Cluster	p
C1	0.004
C2	0.402

Age group 50 - 59

Cluster	p
C1	0.158
C2	0.079
C3	0.758
C4	0.134

Age group 60 - 69

Cluster	p
C1	0.891
**C2**	**<0.001**
C3	0.797
C4	0.643
**C5**	**<0.001**
C6	0.005

Age group 70 - 79

Cluster	p
C1	0.074
C2	0.295
C3	0.63
C4	0.006
C5	0.379
**C6**	**<0.001**

Age group 80+

Cluster	p
C1	0.019
C2	0.279

Supplementary Figure 12. Box plot diagrams for serum glucose values within individual age groups for the extracted clusters are presented. For age groups where more than one cluster has been found, the cluster-specific distribution of measured values is compared with the population distribution. Corresponding p-values are presented in the table below the box plots. The horizontal red and blue lines indicate the upper and lower normal values, respectively. Serum glucose values are measured in millimoles per liter (mmol/L).

SERUM CHOLESTEROL (mmol/L)

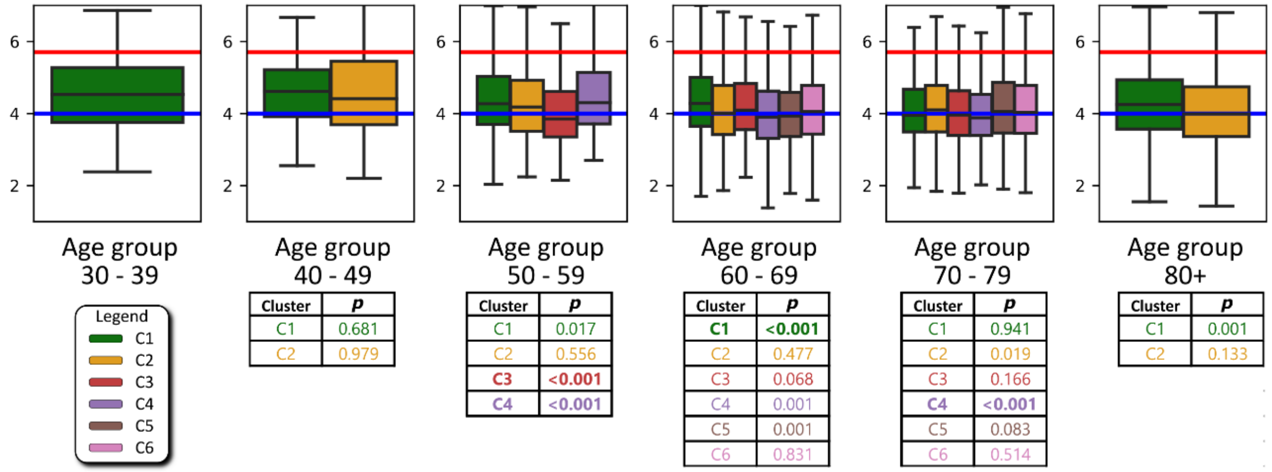

Age group 40 - 49

Cluster	*p*
C1	0.681
C2	0.979

Age group 50 - 59

Cluster	*p*
C1	0.017
C2	0.556
**C3**	**<0.001**
**C4**	**<0.001**

Age group 60 - 69

Cluster	*p*
**C1**	**<0.001**
C2	0.477
C3	0.068
C4	0.001
C5	0.001
C6	0.831

Age group 70 - 79

Cluster	*p*
C1	0.941
C2	0.019
C3	0.166
**C4**	**<0.001**
C5	0.083
C6	0.514

Age group 80+

Cluster	*p*
C1	0.001
C2	0.133

Supplementary Figure 13. Box plot diagrams for serum cholesterol values within individual age groups for the extracted clusters are presented. For age groups where more than one cluster has been found, the cluster-specific distribution of measured values is compared with the population distribution. Corresponding p-values are presented in the table below the box plots. The horizontal red and blue lines indicate the upper and lower normal values, respectively. Serum cholesterol values are measured in millimoles per liter (mmol/L).

SERUM TRIGLYCERIDES (mmol/L)

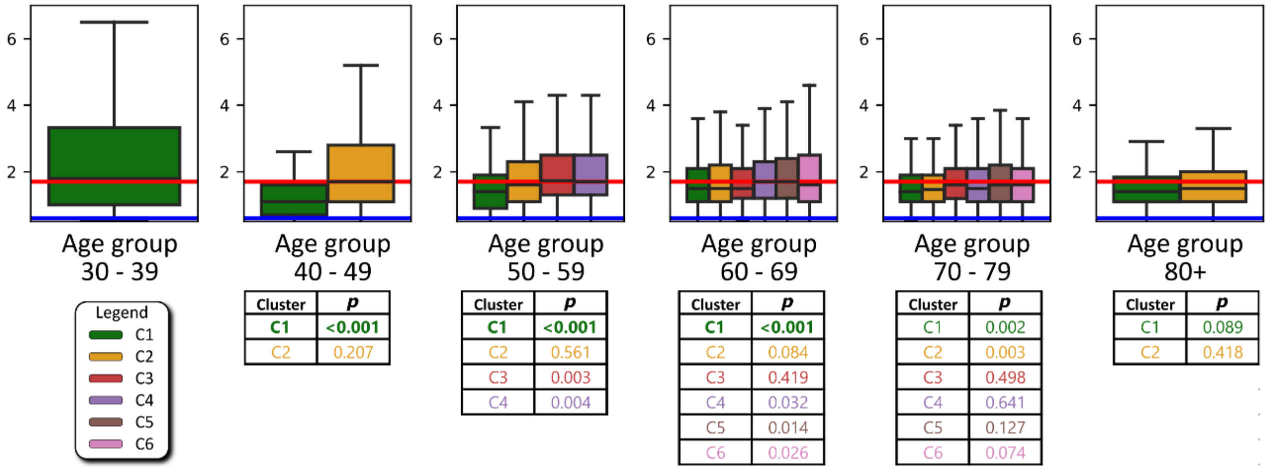

Age group 40 - 49

Cluster	*p*
**C1**	**<0.001**
C2	0.207

Age group 50 - 59

Cluster	*p*
**C1**	**<0.001**
C2	0.561
C3	0.003
C4	0.004

Age group 60 - 69

Cluster	*p*
**C1**	**<0.001**
C2	0.084
C3	0.419
C4	0.032
C5	0.014
C6	0.026

Age group 70 - 79

Cluster	*p*
C1	0.002
C2	0.003
C3	0.498
C4	0.641
C5	0.127
C6	0.074

Age group 80+

Cluster	*p*
C1	0.089
C2	0.418

Supplementary Figure 14. Box plot diagrams for serum triglycerides values within individual age groups for the extracted clusters are presented. For age groups where more than one cluster has been found, the cluster-specific distribution of measured values is compared with the population distribution. Corresponding p-values are presented in the table below the box plots. The horizontal red and blue lines indicate the upper and lower normal values, respectively. Serum triglyceride values are measured in millimoles per liter (mmol/L)

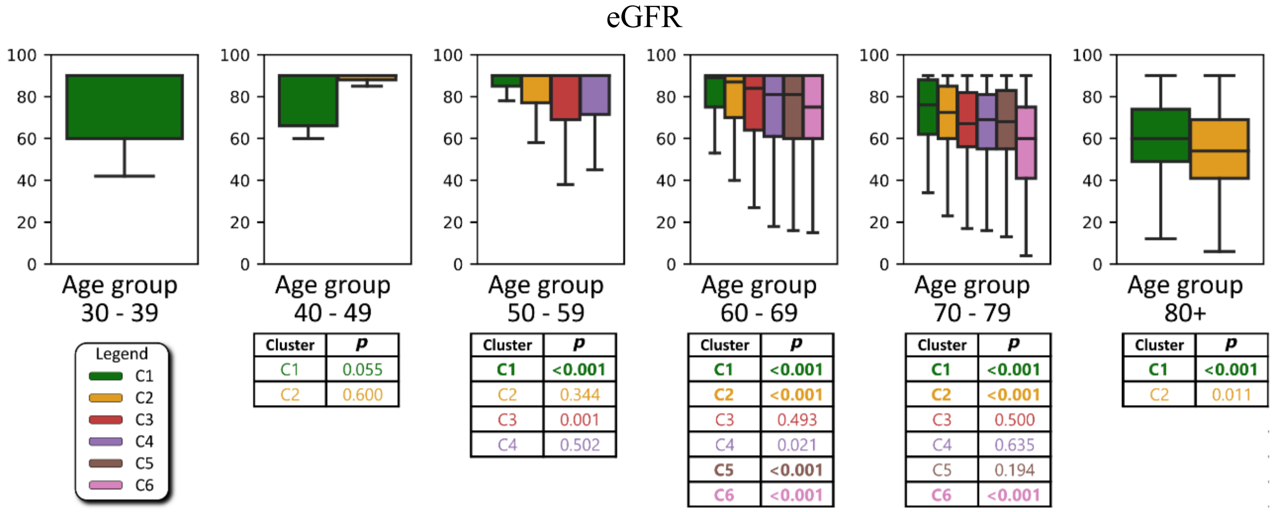

Age group 40 - 49

Cluster	*p*
C1	0.055
C2	0.600

Age group 50 - 59

Cluster	*p*
**C1**	**<0.001**
C2	0.344
C3	0.001
C4	0.502

Age group 60 - 69

Cluster	*p*
**C1**	**<0.001**
**C2**	**<0.001**
C3	0.493
C4	0.021
**C5**	**<0.001**
**C6**	**<0.001**

Age group 70 - 79

Cluster	*p*
**C1**	**<0.001**
**C2**	**<0.001**
C3	0.500
C4	0.635
C5	0.194
**C6**	**<0.001**

Age group 80+

Cluster	*p*
**C1**	**<0.001**
C2	0.011

Supplementary Figure 15. Box plot diagrams for eGFR. values within individual age groups for the extracted clusters are presented. For age groups where more than one cluster has been found, the cluster-specific distribution of measured values is compared with the population distribution. Corresponding *p*-values are presented in the table below the box plots. The horizontal red and blue lines indicate the upper and lower normal values, respectively. The estimated glomerular filtration rate values are measured in the unit's milliliters per minute (mL/min).

We lastly focus on estimating to what extend normal eGFR values are measured in the individual age groups. Typical eGFR values are considered in the range 90 to 120 mL/min. However, even eGFR values above 60 mL/min are still considered as normal. An eGFR below 60 mL/min may be a sign of chronic kidney disease. An eGFR below 15 mL/min is a sign of kidney failure and requires immediate medical attention. Thus, we compute the share of measured eGFR values, discretized into four categories. Category 1 refers to eGFR values in the range 90 to 120 mL/min, category 2 refers to eGFR values in the range 60 to 89 mL/min, category 3 refers to eGFR values in the range 15 to 59 mL/min and category 4 refers to eGFR below 15 mL/min. Results are presented in the Supplementary Figure 16. The chart provides a visual comparison of kidney function across different age groups.

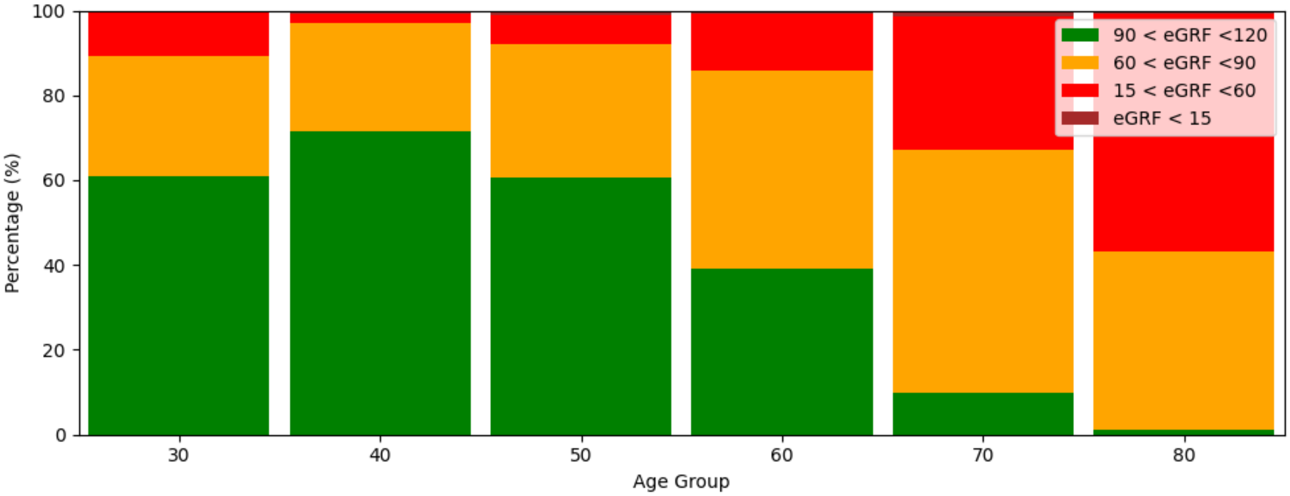

Supplementary Figure 16. Stacked bar chart representing the distribution of estimated glomerular filtration rate (eGFR) values across different age groups. The eGFR values are categorized into four ranges: above 90 mL/min (green), between 60 and 90 mL/min (orange), between 15 and 60 mL/min (red), and below 15 mL/min (brown). Each bar represents an age group, and the height of the colored segments within each bar indicates the share of measured eGFR values within the corresponding range.

The Supporting Table 1 additionally presents the numeric values of the estimated glomerular filtration rate (eGFR) analysis across different age groups. In the youngest age group (30-39 years), most individuals with diabetes (60.8%) had eGFR values above 90 mL/min, indicating optimal kidney function. Only a small fraction (10.8%) had eGFR values between 15 and 60 mL/min, suggesting reduced kidney function, and there were no cases with eGFR values below 15 mL/min. As age increased, we observed a shift in the distribution of eGFR values. In the 40-49 years age group, 71.7% of individuals had eGFR values above 90 mL/min, but this percentage decreased to 60.6% in the 50-59 years age group, and further decreased to 39.1% in the 60-69 years age group. In the oldest age groups (70-79 years and 80+ years), most individuals had eGFR values between 15 and 90 mL/min, indicating reduced kidney function. In the 80+ years age group, only 1.1% of individuals had eGFR values above 90 mL/min, while a significant majority (56.1%) had eGFR values between 15 and 60 mL/min.

Supporting table 1: The percentage of eGFR values categorized into four ranges: above 90 mL/min (category 1), between 60 and 90 mL/min (category 2), between 15 and 60 mL/min (category 3), and below 15 mL/min (category 4).

Age Group	Category 1 ($eGFR > 90 \frac{mL}{min}$)	Category 2 ($60\frac{mL}{min} < eGFR < 90\frac{mL}{min}$)	Category 3 ($15\frac{mL}{min} < eGFR < 60 \frac{mL}{min}$)	Category 4 ($eGFR < 15 \frac{mL}{min}$)
30-39	60.8%	28.4%	10.8%	0.0%
40-49	71.7%	25.5%	2.2%	0.6%
50-59	60.6%	31.6%	6.7%	1.1%
60-69	39.1%	46.9%	13.2%	0.8%
70-79	10%	57.1%	31.6%	1.3%
80+	1.1%	42.1%	56.1%	0.7%

References

1. World Health Organization. International Statistical Classification of Diseases and Related Health Problems (ICD). (2022) https://www.who.int/standards/classifications/classification-of-diseases

2. Zepeda-Mendoza ML, Resendis-Antonio O. "Hierarchical Agglomerative Clustering.," In: Dubitzky W, Wolkenhauer O, Cho K-H, Yokota H, editors. *Encyclopedia of Systems Biology*. New York, NY: Springer New York (2013). p. 886–887 doi: 10.1007/978-1-4419-9863-7_1371

3. Markovič R, Gosak M, Grubelnik V, Marhl M, Virtič P. Data-driven classification of residential energy consumption patterns by means of functional connectivity networks. *Appl Energy* (2019) 242:506–515. doi: 10.1016/j.apenergy.2019.03.134
